# Comprehensive study of anomalous hysteresis behavior in perovskite-based solar cells

**DOI:** 10.1038/s41598-022-19194-5

**Published:** 2022-09-01

**Authors:** Mehran Minbashi, Elnaz Yazdani

**Affiliations:** Department of Physics, Tarbiat Modares University, P.O. Box 14115-175, Tehran, Iran

**Keywords:** Photonic devices, Electronics, photonics and device physics, Semiconductors

## Abstract

Perovskite solar cells (PSCs) have shown remarkable progress with the rapid increase in power conversion efficiency to reach 25.7% over the last few years. However, it is difficult to precisely determine the energy conversion efficiency for PSC, because of anomalous current density-voltage (J–V) hysteresis. Normal J–V hysteresis has been reported in many papers, where the backward scan performance is higher than the forward scan one. In this work, using Drift–Diffusion Modeling, normal hysteretic behavior associated with ion migration with different scanning rates, pre*-*bias voltages*,* and charge-carrier mobility is studied. In addition, the inverted J–V hysteresis by modification of the simulation model, where anions and cations flux towards the transport layers and are accumulated simultaneously on both sides, is achieved. It is also found that the flux parameter values (g_ae_ and g_ch_) play a critical role in the reduction of inverted hysteresis and the efficiency enhancement. It is suggested from the current studies that perovskite interfaces encapsulation, which prevents ions migration, could be of great importance for achieving hysteresis-free PSCs and reliable device characteristics.

## Introduction

Perovskite Solar cells (PSCs) have attracted much attention in recent years due to their outstanding photovoltaic performance^1–10^. Results from many published papers indicate that the chosen measurement protocol has a critical role in data reproducibility^11^. It is not easy to correctly characterize semiconductor material such as perovskite with simultaneous electronic and which led to some confusion and controversy on reported cell performance usually in the form of Current–voltage hysteresis. Various reasons have been proposed by different studies to explain the hysteretic behavior of Perovskite solar cells such as (i) unbalanced charge carrier transport, where hole transport layers (HTLs) and electron transport layers (ETLs) play critical roles in this behavior; (ii) trapping/detrapping of charge carriers; (iii) the ferroelectric property associated with the photoactive perovskite material; and (iv)^13^ ion migration. Reports show that perovskite might be free of ferroelectricity^14,15^. Also, for charge trapping, the response time is about milliseconds which does not contribute to the time scale of hysteresis in J–V measurements^15,16^. Also, MAPbI_3_ perovskite material can dissociate into methylamine ion (MA^+^) and iodide (I^-^), leading to ionic migration. The J–V hysteresis in perovskite solar cells has been mostly attributed to ion migration. It has already been recognized that the hysteresis is influenced by different processing conditions and testing methods^18^. For typical hysteresis (normal hysteresis), the BS performance is higher than the FS one. Recently, anomalous hysteresis (inverted hysteresis) attracted significant attention in PSC, in which the BS results exhibit lower performance than FS under certain circumstances. Almora et al. using drift–diffusion simulation has shown how ion distribution switching depending on the pre-biasing protocol alters charge collection and recombination^19^. Singh et al. has also used the drift–diffusion model and revealed that cation-mediated recombination leads to significant J–V hysteresis^20^. Furthermore, normal (NH) and inverted hysteresis (IH) have been realized by Nemnes et al., in which the pre-polling bias effect on J–V characteristics has been investigated^21^. Despite the number of previous experimental studies, the physical origin of the inverted hysteresis remains unresolved^18^. In this work, the occurrence of normal hysteresis versus scan rates, perovskite mobility, and pre-bias voltages for two models of ions migration; the first model: cation migration, and the second model: anions and cations migration has been studied. Then, the inverted J − V hysteresis by modification of the simulation model, where anions and cations flux towards the transport layers and are accumulated simultaneously on both sides, has also been scrutinized. Two flux parameter values (g_ae_ and g_ch_) have been defined which present a key role in the decrease of inverted hysteresis and the efficiency enhancement.

## Numerical modeling

### Carrier transport equations

The device properties of perovskite-based solar cells have been simulated utilizing the Finite Element Method (FEM) Method^22^. We calculate cell parameters and performance using the numerical solution of the basic semiconductor equations; these equations constitute the Poisson equation Eq. (1), which links the electrostatic potential to total charge density, the continuity equation for holes and electrons [Eqs. (2) and (3)]. Besides, the device performance by considering the Shockley–Read–Hall (SRH) recombination statistics is studied^23–30^.

1 $$\frac{{d}^{2}}{{dx}^{2}}\Phi \left(x\right)=\frac{q}{{\varepsilon }_{0}{\varepsilon }_{r}}\left(p\left(x\right)-n\left(x\right)+{N}_{D}-{N}_{A}+{n}_{t}^{+}-{n}_{t}^{-}\right)$$

2 $$-\left(\frac{1}{q}\right)\frac{{\partial J}_{p}}{\partial x}-{U}_{p}+G=\frac{\partial p}{\partial t}$$

3 $$\left(\frac{1}{q}\right)\frac{{\partial J}_{n}}{\partial x}-{U}_{n}+G=\frac{\partial n}{\partial t}$$

In Eq. (1), parameter $\Phi$ is the electrostatic potential, *q* is the electrical charge, *ε*_*r*_ and *ε*_*0*_ are the relative and the vacuum permittivity, *p* and *n* are hole and electron concentrations, *N*_*D*_ and *N*_*A*_ are charge impurities of donor and acceptor type, ${n}_{t}^{+}$ and ${n}_{t}^{-}$ are hole and electron trap concentrations, respectively. In Eqs. (2) and (3); *J*_*n*_ and *J*_*p*_ are the electron and hole current densities. *G* is the generation rate and its calculation has been explained in the Supplementary Information (SI). The equations for ionic species migration, anions (*a*^−^) and cations (*c*^+^), are coupled with drift–diffusion equations (Eqs. (1–3)) as follows:

4 $$- \left( \frac{1}{q} \right)\frac{{\partial J_{{c^{ + } }} }}{\partial x} = \frac{\partial c}{{\partial t}}$$

5 $$\left( \frac{1}{q} \right)\frac{{\partial J_{{a^{ + } }} }}{\partial x} = \frac{\partial a}{{\partial t}}$$

In addition, the Poisson equation by including the anions (*a*^−^) and cations (*c*^+^) concentration is modified to:

6 $$\frac{{d}^{2}}{{dx}^{2}}\Phi \left(x\right)=\frac{q}{{\varepsilon }_{0}{\varepsilon }_{r}}\left(p\left(x\right)-n\left(x\right)+{N}_{D}-{N}_{A}+{n}_{t}^{+}-{n}_{t}^{-}+({c}^{+}-{a}^{-})\right)$$

The neutrality condition is satisfied to the entire perovskite by the following equation:

7 $$1/l\iint {a}^{-}\left(x\right)dx=1/l\iint {c}^{+}\left(x\right)dx={N}_{i}$$

where $l$ is the total thickness of perovskite, and ${N}_{i}$ is ion density.

In the perovskite interfaces with hole and electron transport layers, electronic and ionic carriers can tunnel to the ETL and HTL layers. For this mean, the WKB approximation has been considered for interfaces to satisfy the charge carriers tunneling effect. All details about the used WKB equations in simulation have been clarified in the SI (Fig. S1).

### Boundary condition for ionic transport

#### No flux of ions into the ETL and HTL

Two types of boundary conditions for ionic carriers have been examined in our simulations. In the first case, there is no flux of anions and cations across the perovskite/ETL and perovskite/HTL. This conventional definition for boundary condition is being used in many simulation^15,31^ studying are the set of equations reads as follows:

8 $$n.{\mathrm{J}}_{a}=0$$

9 $$n.{\mathrm{J}}_{c}=0$$

10 $${\mathrm{J}}_{a}={q\mu }_{a}aE+{qD}_{a}\frac{\partial a}{\partial x}$$

11 $${\mathrm{J}}_{c}={q\mu }_{c}cE-{qD}_{c}\frac{\partial c}{\partial x}$$

where n is the normal vector, J_a_, and J_c_ are anion and cation current density, respectively. D_a_, and D_c_ are anion and cation diffusion coefficients, respectively.

#### Flux of ions into the ETL and HTL

To provide much more accurate simulation results compared to experimental outcomes, new boundary conditions that the anions and cations can flux through the perovskite interfaces have been defined. We believe that this proposed model can obtain some anomalous behaviors such as High Inverted Hysteresis (HIH), which has been proven by experiments. There are very few papers, which clarified the occurrence of the HIH by J–V characterization conditions such as negative/reverse, and high pre-bias (pre-conditioning) voltage in their experiments and simulations^21,32^. However, the manifestation of HIH has been reported in some other experimental conditions^33–37^ and has not yet been approved by simulation modeling.

The defined equations are as follows:

12 $$n.{\mathrm{J}}_{a}=q({g}_{a}-{Q}_{a}a)$$

13 $$n.{\mathrm{J}}_{c}=q({g}_{c}-{Q}_{c}c)$$

where ${g}_{a}$, and ${g}_{c}$ are the boundary flux of anion and cation, respectively. ${Q}_{a}$ , and ${Q}_{c}$ are the anion, and cation boundary absorption velocities. The values of ${g}_{a}$, and ${g}_{c}$ are in the range of:

14 $${{g}_{a},g}_{c}\ll {d}_{perovskite}.{G}_{avpsk}$$

where ${d}_{perovskite}$ is the thickness of perovskite, and ${G}_{avpsk}$ is the average generation rate of perovskite.

## Results and discussion

### Validation results

**Figure 1:**
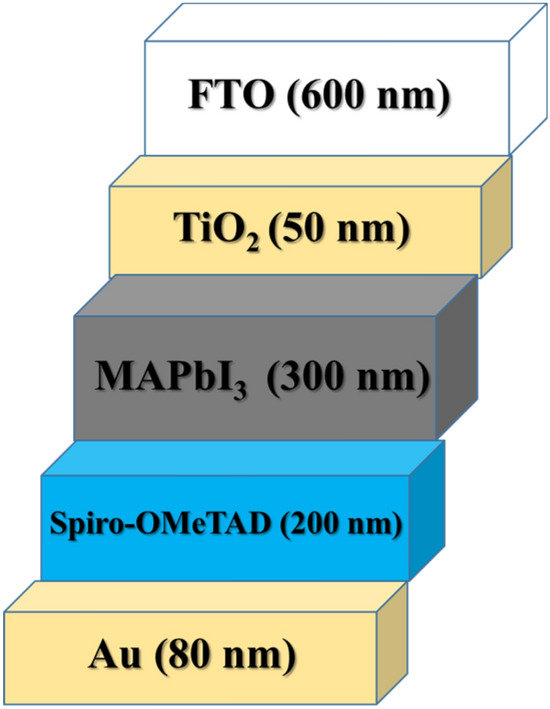
Schematic of solar cell for the simulation.

The one-dimensional drift–diffusion model^38^ is modified to simulate the hysteresis effect of ion migration. We set up a perovskite solar cell according to the experimental study^39^ with a 300 nm MAPbI_3_ absorber layer, a 200 nm hole transport layer (HTL), and a 50 nm electron transport layer (ETL) using TiO_2_ and Spiro-OMeTAD, respectively. The device structure is shown in Fig. 1.

**Figure 2:**
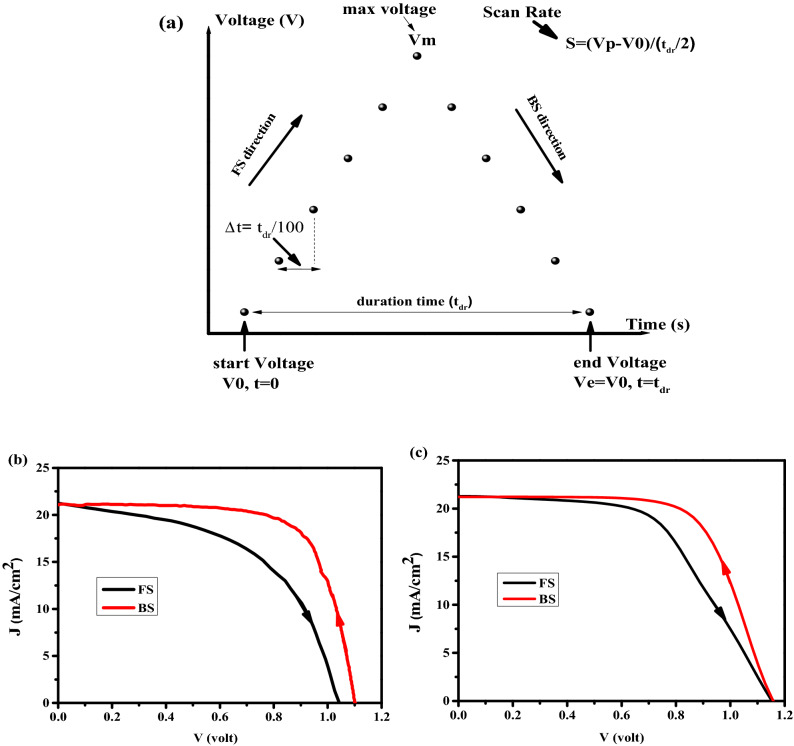
(**a**) Scanning voltage protocol for the simulation. J–V characteristics, for (**b**) experimental, and (**c**) simulation. The scan rate was 100 mV/s for both cases.

All model parameters are shown in Tables 1 and 2. The cell structure is considered FTO/ TiO_2_/ CH_3_NH_3_PbI_3_/spiro-OMeTAD/Au as shown in Fig. 1. The scanning protocol was shown in Fig. 2a, in which V_0_ = 0 V and V_m_ = 1.2 V. In this study, the validation of the simulation results have been carried out by the experimental data. The current–voltage characteristics (J–V) (Fig. 2b,c) show a good compromise between the experimental data and the simulated one.

**Table 1:** List of electro-optical parameters used in carrier transport simulation.

Parameter and units	TiO_2_ (ETL)	MAPbI_3_	Spiro-Ometad (HTL)
Thickness (nm)	50 [exp]	300 [exp]	200 [exp]
Electron affinity (eV)	4.1^32^	3.6 [fitting]	1.9^32^
Bandgap (eV)	3^32^	1.52 [fitting]	3^32^
Dielectric permittivity (relative)	31^32^	18^32^	3^32^
CB effective density of states (cm^-3^)	1 × 10^20^^32^	5.0 × 10^18^^32^	1 × 10^20^^32^
VB effective density of states (cm^−3^)	1 × 10^20^^32^	5.0 × 10^18^^32^	1 × 10^20^^32^
Electron mobility (cm^2^/Vs)	10^–2^^32^	2^32^	2^32^
Hole mobility (cm^2^/Vs)	2^32^	2^32^	10^–232^
Shallow uniform donor density N_D_ (cm^−3^)	1.0 × 10^18^^32^	0^32^	0^32^
Shallow uniform acceptor density NA (cm^−3^)	0^32^	0^32^	1.0 × 10^18^^32^
Absorption constant A (1/cm eV^(1/2)^)	1.0 × 10^4^^40^	1.15 × 10^5^	3.0 × 10^4^^32^
Absorption constant B (eV^(1/2)^/cm)	0	0	0
Electron thermal velocity (cm/s)	1.0 × 10^7^^40^	1.0 × 10^7^^40^	1.0 × 10^7^^40^
Hole thermal velocity (cm/s)	1.0 × 10^7^^40^	1.0 × 10^7^^40^	1.0 × 10^7^^40^
Electron Life Time (ns)	5^41^	100^32^	5^41^
Hole Life Time (ns)	5^41^	100^32^	5^41^

*exp: experiment.

**Table 2:** List of parameters related to ion migration^20,42–45^.

Parameter and units	MAPbI_3_
Anion mobility (cm^2^/Vs)	1 × 10^–11^
Cation mobility (cm^2^/Vs)	1 × 10^–11^
Anion density Na(cm^−3^)	9 × 10^16^
Cation density Nc (cm^−3^)	9 × 10^16^
${g}_{a}$(1/m^2^ s)	10^15^–5 × 10^16^
${g}_{c}$(1/m^2^ s)	10^15^–5 × 10^16^
${Q}_{a}$(m/s)	0
${Q}_{c}$(m/s)	0

Data are taken from valid references.

Additionally, the primary data for the electron–hole lifetime (defect properties), and ion properties have been shown in Tables 1, and 2, respectively.

Effect of No-Ion also is investigated in SI (Fig. S2 and Tables S1,2).

### Time-dependent study of PSCs

#### Model 1: Scan-dependent hysteresis by considering cation migration

Hysteresis index can be defined as:

15 $$HI=\frac{{PCE}_{BW}-{PCE}_{FW}}{{PCE}_{BW}}$$

**Figure 3:**
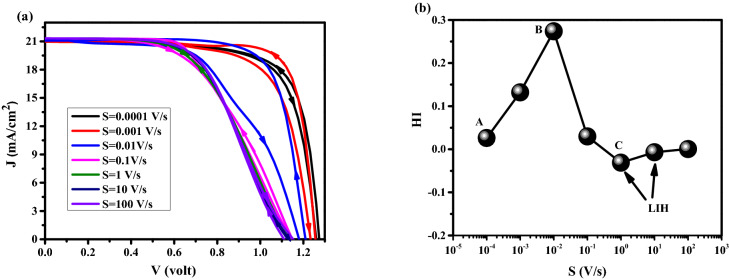
(**a**) Effect of different scan rates on J–V hysteresis, and (**b**) hysteresis index vs scan rate for perovskite solar cell.

**Figure 4:**
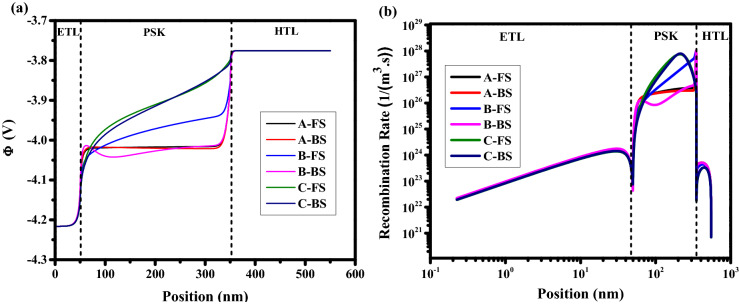
Effect of different scan rates on (**a**) electric potential, and (**b**) recombination rate for holes (equal to electrons). Graphs were taken at V = 1 V (t = 1.008 × 10^4^ s, 1.008 × 10^2^ s, and 1.008 s which means V = 1 V in FS for S = 10^–4^ V/s, 10^–2^ V/s, and 1 V/s respectively, and t = 1.792 × 10^4^ s, 1.792 × 10^2^ s, and 1.792 s which means V = 1 V in BS for S = 10^–4^ V/s, 10^–2^ V/s, and 1 V/s respectively), and the illumination of light condition.

For $\mathrm{HI}>0$ there is Normal hysteresis (NH) and for $\mathrm{HI}<0$ there is inverted hysteresis (IH). For IH, we have V_oc_ decay, J_sc_ decay, FF decay, and S-shaped type. To study the impact of ions type, initially, the dynamic positive ions (cations) are considered in the active layer, and uniformly distributed negative ions are considered stationary. As one can realize from the J–V analysis in Fig. 3a, with scan rates from 10^–4^ to 10^–3^ V/s (low scan rates), the lowest hysteresis index (HI = 0.0260) and the highest cell efficiency are obtained. At scan rate of 10^–2^ V/s (intermediate scan rate), the J–V characteristics show the highest hysteresis index (HI = 0.2738) compare to the results with low scan rates. For the scan rates between 10^–1^ and 10^2^ (high scan rates), lower hysteresis indexes (HI = 6.0537 × 10^–4^) are obtained while the averaged cell efficiency is dropped to about 13.2% which is less than the efficiencies attained with low scan rates. Three points have been labeled by letters A, B, and C in Fig. 3b represent the slow scan rate (long scanning time—high efficiency), medium scan rate (medium scanning time), and high scan rates (short scanning time—low efficiency), respectively. At point A, the scanning time of the applied voltage is long enough to allow the cations to move sufficiently fast compared to the voltage sweeping and the cation distribution remains close to quasi-equilibrium, causing the electric potential at FS and BS to be nearly the same^42^. Scan-independent hysteresis origins from the similar current behavior for both scan directions, which leads to low HI. In this case, we have a strong electric potential (Fig. 4a), which promotes the charge extraction from the PSK layer, and decreases the recombination rate in PSK (Fig. 4b), causing the high efficiency. For point B (intermediate scan rate), the history of the scan influences the cation distribution, internal fields and current^42^ that exhibit obvious hysteresis. The large difference between FS and BS in the electric potential and recombination rate has caused severe hysteresis. At point C, a strong S-shape in the J–V can be seen that is mainly associated with the screening of the built-in field due to the accumulation of cations at the interface between the PSK and the transport layers and enhancing the recombination rate and decreasing the electric field which results in low efficiency in comparison with A and B. At such high scan rates, the ions cannot follow the change in the applied voltage (cation motion is not fast enough), which leads to a negligible change in electric potential and recombination rate in both FS and BS directions also we have low inverted hysteresis (LIH) at this point.

Effects of electron–hole mobility and pre-bias voltage on model 1 are available in the supplementary information (SI) (Figs. S3–6 and Tables S3–8).

### Model 2: Effect of cations and anions on perovskite solar cell

#### Effect of different scan rates

**Figure 5:**
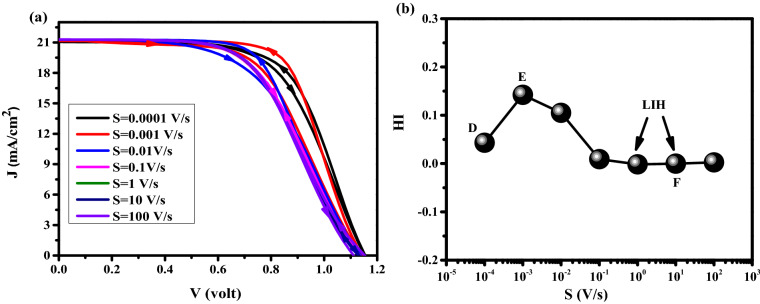
(**a**) Effect of different scan rates on J–V hysteresis, and (**b**) hysteresis index vs scan rate for perovskite solar cell.

**Figure 6:**
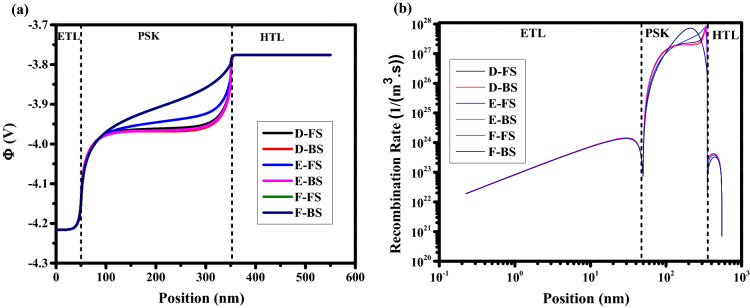
Effect of different scan rates on (**a**) electric potential, and (**b**) total recombination rate. Graphs were taken at V = 1 V (t = 1.008 × 10^4^ s, 1.008 × 10^3^ s, and 1.008 × 10^–1^ s which means V = 1 V in FS for S = 10^–4^ V/s, 10^–3^ V/s, and 10 V/s respectively, and t = 1.792 × 10^4^ s, 1.792 × 10^3^ s, and 1.792 × 10^–1^ s which means V = 1 V in BS for S = 10^–4^ V/s, 10^–3^ V/s, and 10 V/s respectively), and the illumination of light condition.

In the previous section, only the cations were considered in the perovskite film. A real perovskite cell can consist of both positive (cations) and negative (anions) ions within the perovskite film. As can be seen in Figs. 5a,b, for scan rate of 10^–4^ (low scan rate) and less (which has not been shown here) the low HI of 0.0430 with high efficiency is achieved. At scan rates between 10^–3^ and 10^–2^ V/s (intermediate scan rate), the highest HI (> 0.14) is observed. In the range of 10^–1^ to 10^2^ (high scan rates), small HI (< -2.98 × 10^–4^) with low averaged efficiency, which is less than the efficiencies for the case of low scan rates are achieved. Similar to the previous section, three points D, E and F are chosen to represent the slow scan rate (long scanning time—high efficiency), the medium scan rate (medium scanning time), and the large scan rates (short scanning time—low efficiency), respectively. At point D, the long scanning time allows anions and cations to move sufficiently fast with respect to the voltage sweeping that they can easily follow the change in the applied bias voltages. In addition, ions migration into the transport layers leads to almost unchanged electrical potential and recombination rate in the forward and backward scan directions. Ion distribution remains close to quasi-equilibrium. Accordingly, the stronger electric potential and lower recombination rate from Fig. 6a,b, respectively, could explain the high efficiency of the cells in this condition. At point E (intermediate scan rate), cations and anions distribution is influenced by the history of scanning in the FS direction which causes a stronger electric field and weaker recombination rate in the BS direction. This large difference builds severe HI. At point F, a strong S-shape is observed in the J–V characteristic that originates from the screening of the built-in field due to the ions migration towards interfaces between HTL/PSK and ETL/PSK, respectively^20,42,46,47^. This effect decreases the strength of electric potential and increases the recombination rate in the PSK layer, which leads to low efficiency. Low HI is due to the low sensitivity of ions (ions not fast enough) to the applied voltage variations causing a poor change in the recombination rate and electric potential in both directions^20,42^.

#### Perovskite mobility effect on the cell performance

**Figure 7:**
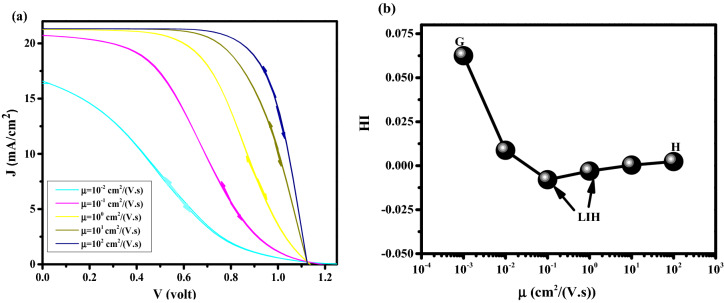
(**a**) Effect of different perovskite mobility on J–V hysteresis, and (**b**) hysteresis index vs mobility for perovskite solar cell.

**Figure 8:**
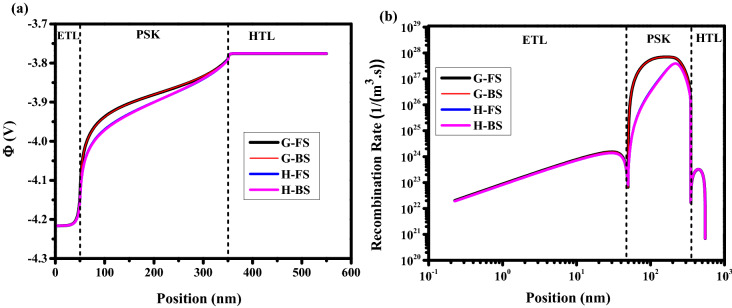
Effect of different mobility on (**a**) electric potential, and (**b**) total recombination rate. Graphs were taken at S = 1 V/s, V = 1 V (t = 1.008 s which means V = 1 V in FS, and t = 1.792 s which means V = 1 V in BS), and the illumination of light condition.

Mobility of perovskite is an important factor for charge carrier transport and high-efficiency achievement. In this section, the effect of perovskite mobility on J–V characteristics and HI parameters is investigated. However, the perovskite mobility has a low effect on HI, but it has an important role in efficiency enhancement and HI decrement, simultaneously. As is shown in Fig. 7a, by increasing the perovskite mobility from 10^–2^ cm^2^/V.s to 10^2^ cm^2^/V.s, the cell efficiency is increasing while the HI decreases (Fig. 7b). To get more insight into the physics of observed results, we indicated two points, G (High HI and Low efficiency), and H (Low HI and High efficiency). As illustrated in Fig. 8 for point G, the electric potential is weak (Fig. 8a) and the recombination rate is high (Fig. 8b) in the middle of PSK. This can be explained by the ions screening effect which is caused by poor charge extraction due to the low perovskite layer mobility. For point H, a lower recombination rate in comparison with point G where the efficiency is high is obtained. Furthermore, for point H, the difference between the electric potentials and the recombination rates in FS and BS directions due to the ionic response produces more HI.

#### Effect of different pre-bias

**Figure 9:**
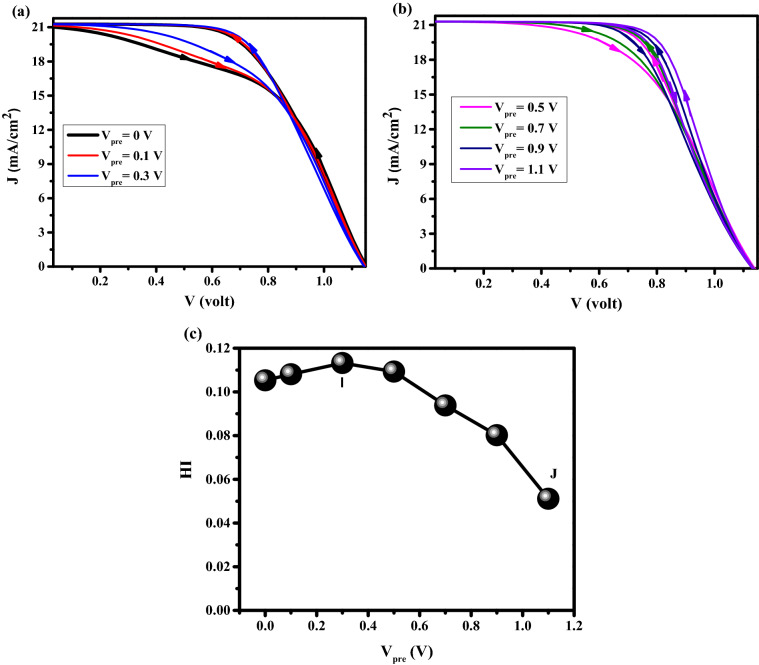
Effect of different pre-bias on J–V characteristics for (**a**) pre-bias voltage = 0 V, 0.1 V, and 0.3 V, and (**b**) pre-bias voltage = 0.5, 0.7 V, 0.9 V, and 1.1 V. (**c**) hysteresis index vs pre-bias voltage. Graphs were taken at S = 1 V/s. All pre-biases were carried out in 10 s.

**Figure 10:**
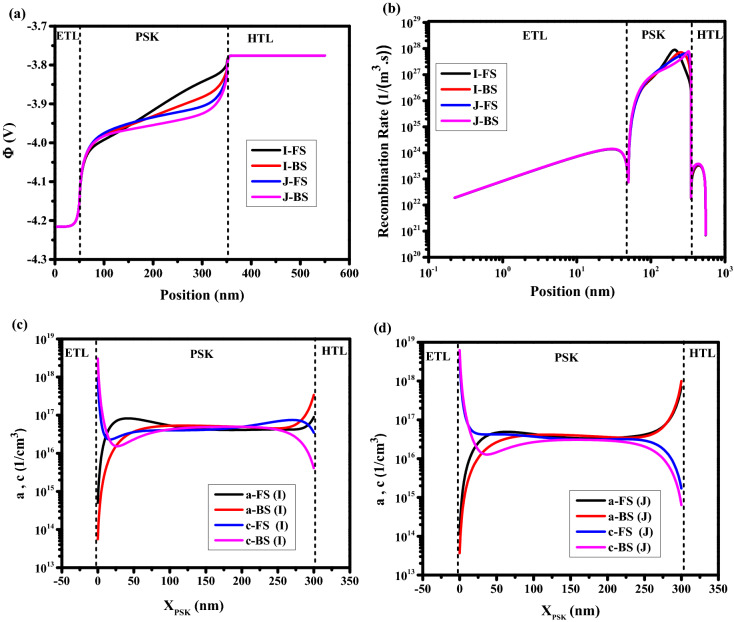
Effect of different pre-bias on (**a**) electric potential, and (**b**) total recombination rate. Anion and cation density distribution in perovskite for (**c**) point I, and (**d**) point J. Graphs were taken at S = 1 V/s, V = 1 V (t = 1.008 s which means V = 1 V in FS, and t = 1.792 s which means V = 1 V in BS), and the illumination of light condition.

Figure 9a,b shows the J–V characteristics versus the pre-bias voltage (Vpre), by increasing Vpre, it is obvious from Fig. 9a,b,c that the cell efficiency is increased and HI is being decreased. Moreover, an S-shaped J–V curved is obtained for low pre-bias voltage (a point I) that originated from S-shaped electric potential, which is clear from Fig. 10a. From the point I to J, the appearance of the recombination peak in Fig. 10b is changing from the middle to the interface region. It means that the ions accumulate from the middle to the interface. The high ion density in the middle of PSK (a point I) reduces the carrier transport which leads to lower electric potential and higher recombination rate in comparison with point J (Fig. 10a,b). The density distribution of anions and cations for the above-mentioned conditions I and J are shown in Fig. 10c,d for the FS and BS directions. It can be realized that there is a big difference between anions and cations density distributions in FS and BS directions for the point I with higher HI with respect to point J. At point J, the high voltage pre-bias lets more anions and cations migrate from the middle to interfaces of HTL/PSK, and ETL/PSK, respectively, which facilitates the carrier transport and decreases the recombination in the large area of the perovskite (Fig. 10b). It can be concluded that the more symmetric the distribution of anions and cations with respect to the center, the higher the efficiency can be reached, and the closer these symmetrical distributions are in two directions of FS and BS, the less hysteresis we will see. Tables S9-14 show the values of J–V characteristics, and HI.

#### Effect of ion flux to ETL and HTL on perovskite solar cell

**Figure 11:**
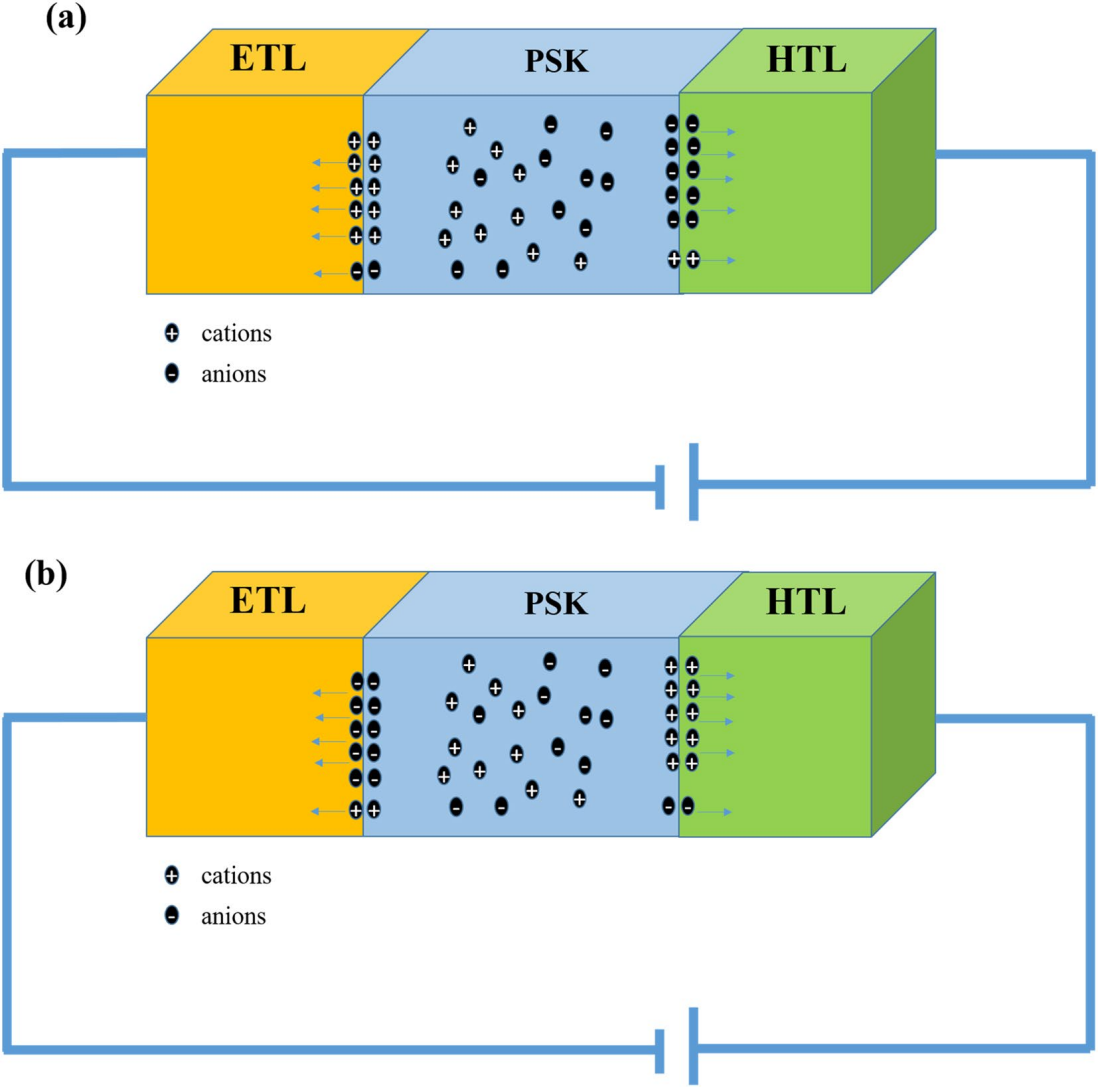
Schematic of the flux of (**a**) anions and cations to HTL and ETL, respectively, and (**b**) anions and cations to ETL and HTL, respectively.

There are only a few studies that considered the ion migration in perovskite-based solar cells in time-dependent modeling. In addition, to the best of our knowledge, the ions flux from the active layer to the transport layers has not also been considered in their calculations. In the experimental condition, the flux of ions to ETL, and HTL are known as important reasons for corrosion and perovskite degradation^48^. We define new equations (Eqs. (12–14)) that consider the ion flux through the ETL and HTL layers. A schematic of anions, and cations diffusion to the ETL, and HTL are shown in Fig. 11a,b, respectively.

#### Flux of anions to HTL and cations to ETL

**Figure 12:**
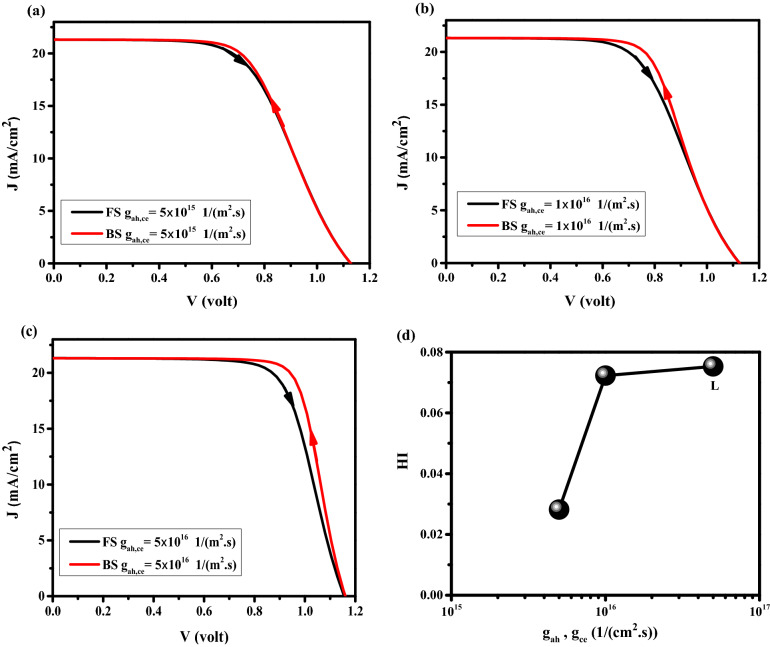
Effect of different flux (g_ah_, and g_ce_) on J–V hysteresis, for (**a**) g_ah_ = g_ce_ = 1 × 10^15^ 1/(m^2^ s), (**b**) g_ah_ = g_ce_ = 5 × 10^15^ 1/(m^2^ s), and (**c**) g_ah_ = g_ce_ = 1 × 10^16^ 1/(m^2^ s). (**d**) HI vs g_ah_ = g_ce_.

**Figure 13:**
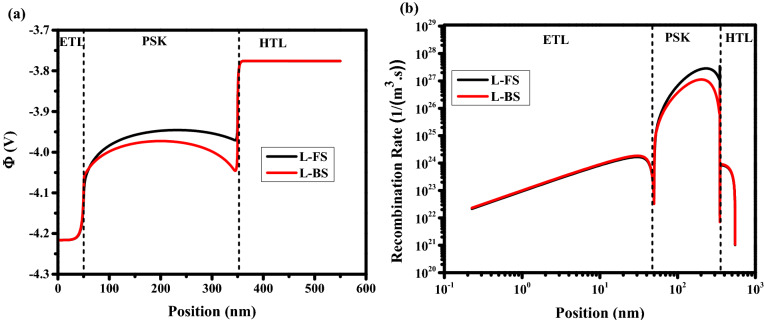
Effect of anion and cation flux to HTL and ETL, respectively (**a**) electric potential, and (**b**) total recombination rate. Graphs were taken at S = 1 V/s, V = 1 V (t = 1.008 s which means V = 1 V in FS, and t = 1.392 s which means V = 1 V in BS), and the illumination of light condition.

The effect of anion and cation flux to HTL, and ETL, respectively, on J–V hysteresis, is investigated. Figures 12a–d shows the effect of different values of g_ah_ (anion flux to HTL), g_ce_ (cation flux to ETL) on J–V characteristics and HI. The values of g_ae_ (anion flux to ETL) and g_ch_ (cation flux to HTL) are 10% of g_ce_, and g_ah_. By increasing g_ah_, and g_ce_, HI is increased and NH has been observed. At point L, The barrier is formed in HTL/PSK energy level due to anions movement towards the HTL that photogenerated carriers accumulate at the barrier under FS compare to BS^18^. This results in a decrement of the charge transport and electric potential, producing more recombination under FS (Fig. 13a,b)^18^. The anion flux to HTL has a strong effect on unfavorable band offset which result in HI increment, but in general, enhancing the cation flux to the ETL causes a reduction in surface recombination rate at ETL/PSK, and ETL regions (Fig. 13b) and result in FF and efficiency increment (Table S15.) at point L in comparison with other cases. The high difference between the electric potentials and recombination rates in FS and BS generates a high HI.

The values of J–V characteristics and HI are available in Tables S15 and S16, respectively.

#### Flux of anions to ETL and cations to HTL

**Figure 14:**
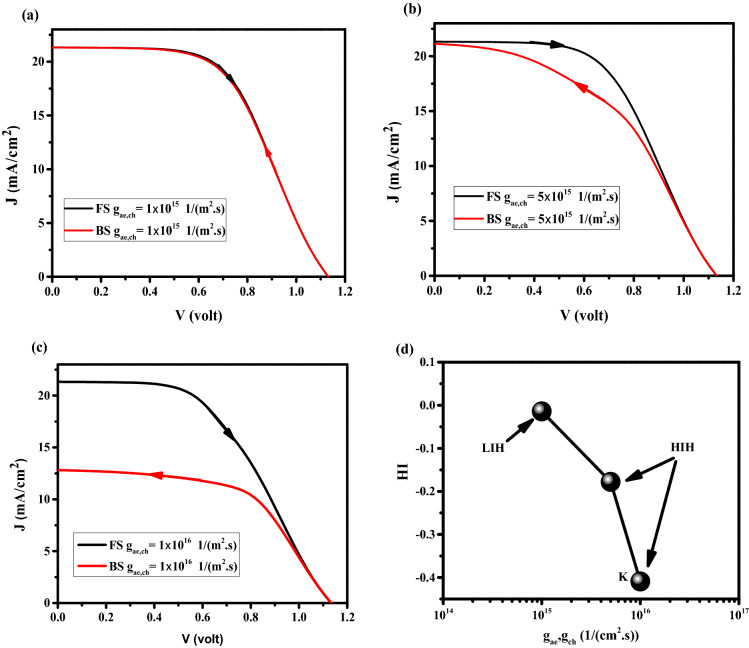
Effect of different flux (g_ae_, and g_ch_) on J–V hysteresis, for (**a**) g_ae_ = g_ch_ = 1 × 10^15^ 1/(m^2^ s), (**b**) g_ae_ = g_ch_ = 5 × 10^15^ 1/(m^2^ s), and (**c**) g_ae_ = g_ch_ = 1 × 10^16^ 1/(m^2^ s). (**d**) HI vs g_ae_ = g_ch_.

**Figure 15:**
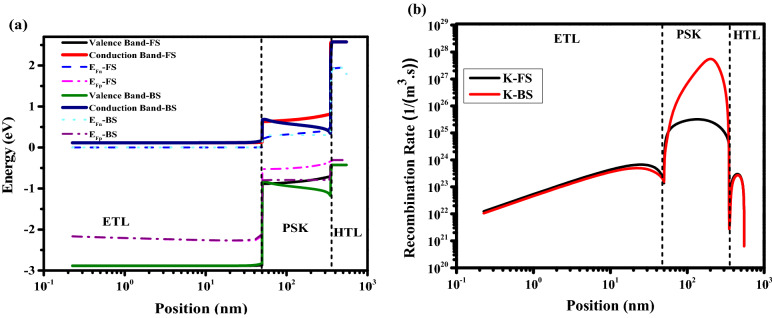
Effect of cation and anion flux to HTL and ETL, respectively (**a**) band structure, and (**b**) total recombination rate. Graphs were taken at S = 1 V/s, V = 0.3 V (t = 0.312 s which means V = 0.3 V in FS, and t = 2.088 s which means V = 0.3 V in BS), and illumination of light condition.

In Fig. 14a–d the effect of anion and cation flux into ETL (g_ae_) and HTL (g_ch_), respectively, is investigated (the values of g_ah_ and g_ce_ were 10% of g_ch_, and g_ae_). By increasing the amount of flux, different configurations of HIH such as decay on the FF, J_sc_ , and S-shaped are observed. Band structure and recombination rate are shown in Fig. 15a,b for FS and BS at V = 0.3 V under illumination for point k. In Fig. 15a, the conduction band and valence band drop to down in BS which prevents the electron carriers to move from PSK to ETL, and the movement of the hole carriers from PSK to HTL, respectively. This leads to low Jsc, FF, and efficiency. This large band offset between BS and FS results in occurring HIH. Consequently, the recombination rate is significantly enhanced in BS (shown in Fig. 15b) and it generates HIH. Cation and anion concentration animation videos have been shown in SI for times 0 to 2.4 s (V1,2 page 25 SI), which show the cation and anion accumulation enhanced in HTL, and ETL interfaces, respectively, create barriers at the carrier transport interfaces, decreasing carrier transfer, resulting in more recombination, low efficiency and high IH. The snapshots of the V1 and V2 are available on pages 26–31 SI. The values of J–V characteristics and HI are available in Tables S16 and S17, respectively. Dark J–V is plotted in Fig. S7. Figs. S8-13 show the carriers and Ionics distribution at different times and voltages.

## Conclusion

We have studied the normal and inverted hysteresis behavior of perovskite solar cells due to ion migration phenomena by varying the hysteresis-related parameters such as scan rate, charge carrier mobility, and pre-bias voltages. Also, we extend the drift–diffusion model by introducing new equations related to the ionic flux. The results show that the long scanning time (low scan rate) lets the ions move fast enough that the distribution of the ions remains close to the quasi-equilibrium, which results in hysteresis-free and high-efficiency performance. Choosing the middle scan rate does not meet the demand to get a hysteresis-free device anymore. High mobility of perovskite supports the facilitating transport of the carriers, increases efficiency, and decreases HI. Increasing the pre-bias voltages, lets more anions and cations migrate from the middle to interfaces of HTL/PSK, and ETL/PSK, respectively, which facilitates the carrier transport and decreases the recombination perovskite layer which results in the efficiency enhancement and HI decrement. In the final step, by considering the ionic flux to ETL and HTL, the high inverted hysteresis, Jsc decay, FF decay, and S shape condition of some types of PSK devices were clarified. Our results show that the higher values of g_ae_ and g_ch_ lead to the HIH and low efficiency. The flux of ions physics also explains the effect of ions on perovskite layer degradations, and corrosion in perovskite solar cell devices, which are important factors instability of these devices. We suggest the interface passivation and capsulation with different experimental methods to achieve high efficiency, hysteresis-free, and stable PSK solar cell devices.

## Supplementary Information

Supplementary Information 1.

Supplementary Information 2.

Supplementary Information 3.

## Data Availability

The data that support the findings of this study are available from the corresponding author, [E.Y.], upon reasonable request.
