# Supplementary material for: Comprehensive study of anomalous hysteresis behavior in perovskite-based solar cells
Source: Sci Rep. 2022 Sep 1;12:14916. doi: 10.1038/s41598-022-19194-5 (PMC9436975; doi:10.1038/s41598-022-19194-5)
Supplement: Supplementary file 1 — Supplementary Information 1. [file 41598_2022_19194_MOESM1_ESM.pptx]

## Slide 1
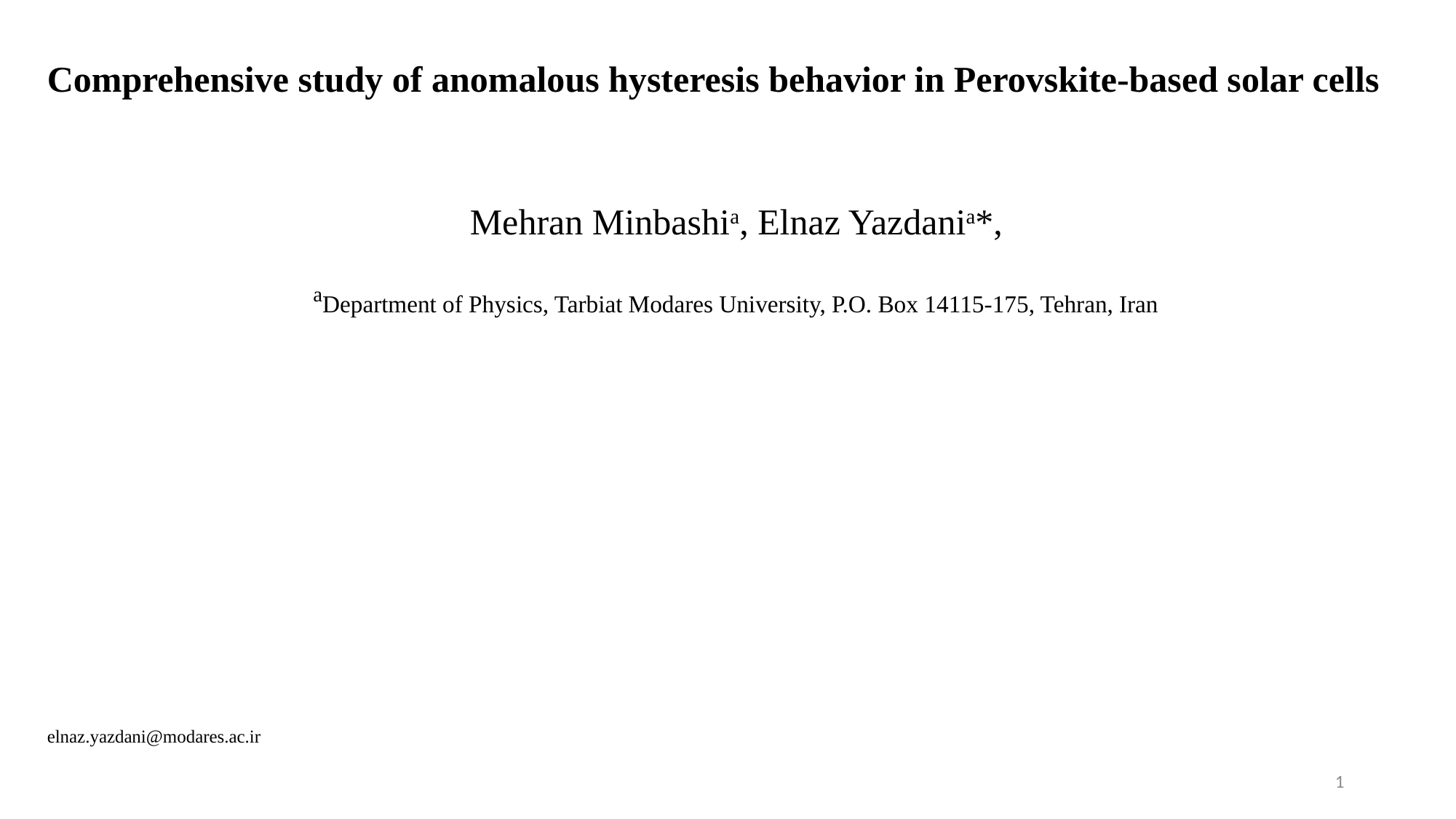

Comprehensive study of anomalous hysteresis behavior in Perovskite-based solar cells
Mehran Minbashia, Elnaz Yazdania*,
aDepartment of Physics, Tarbiat Modares University, P.O. Box 14115-175, Tehran, Iran
elnaz.yazdani@modares.ac.ir
1

## Slide 2
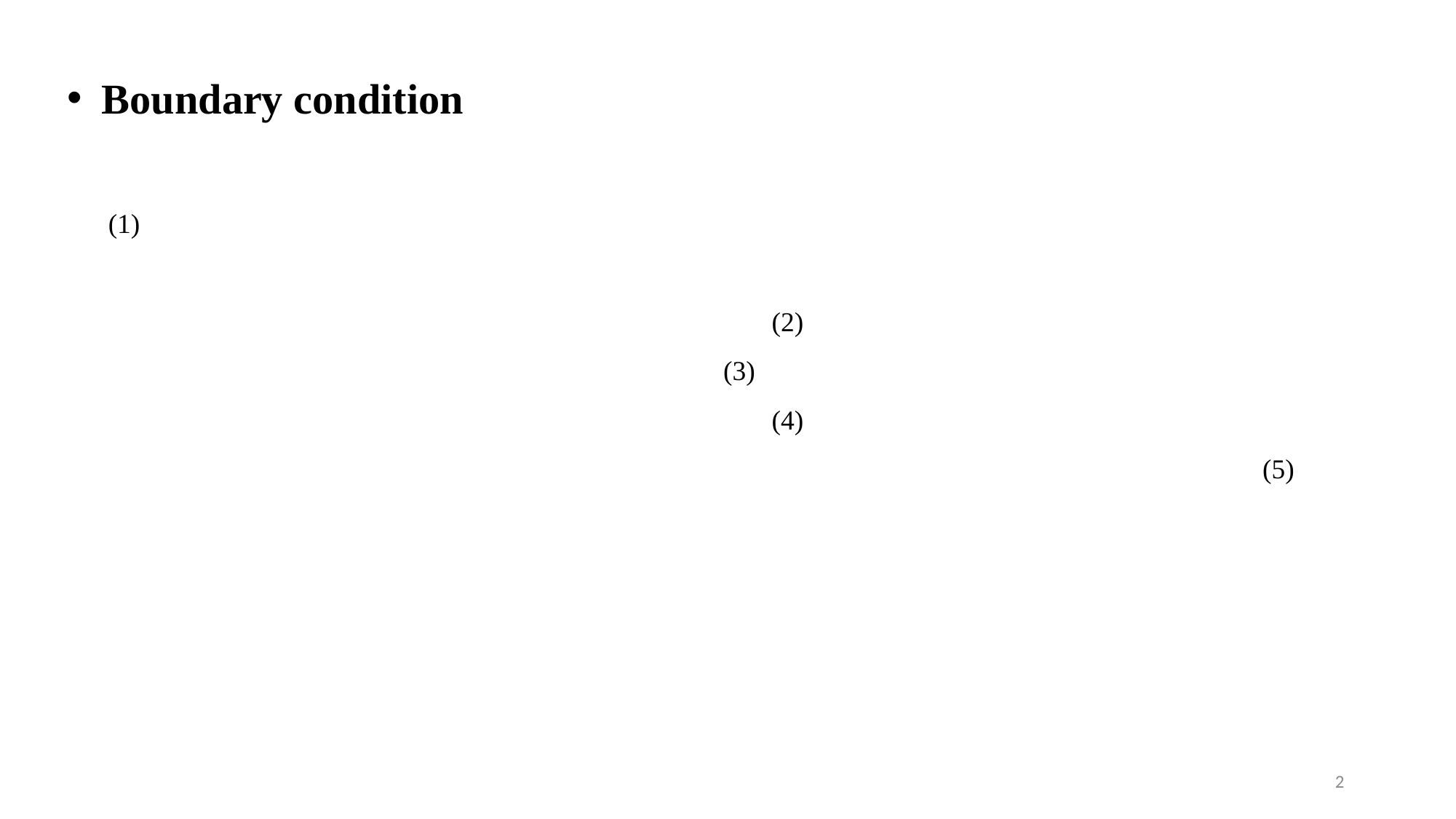

Boundary condition
2

## Slide 3
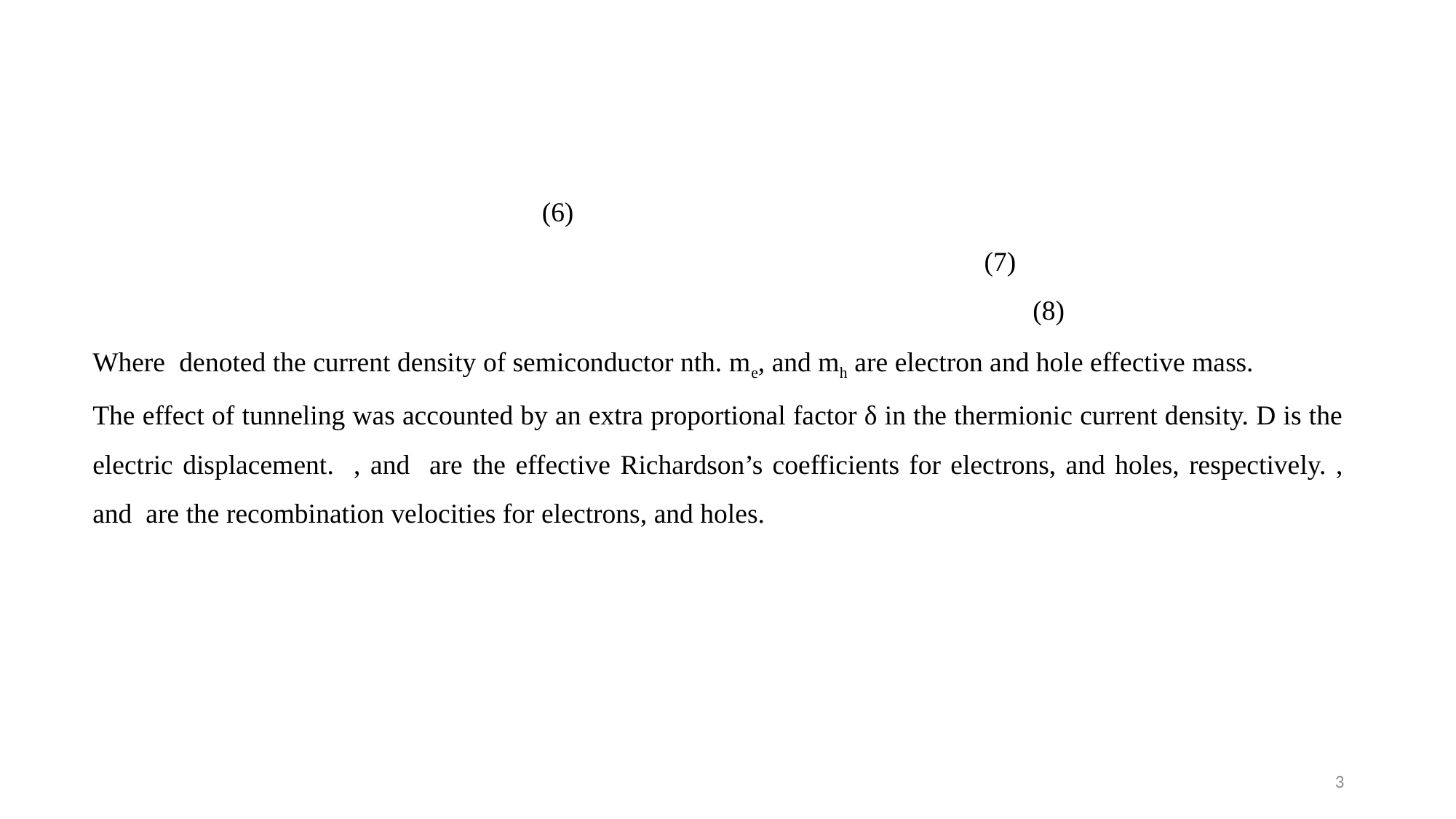

3

## Slide 4
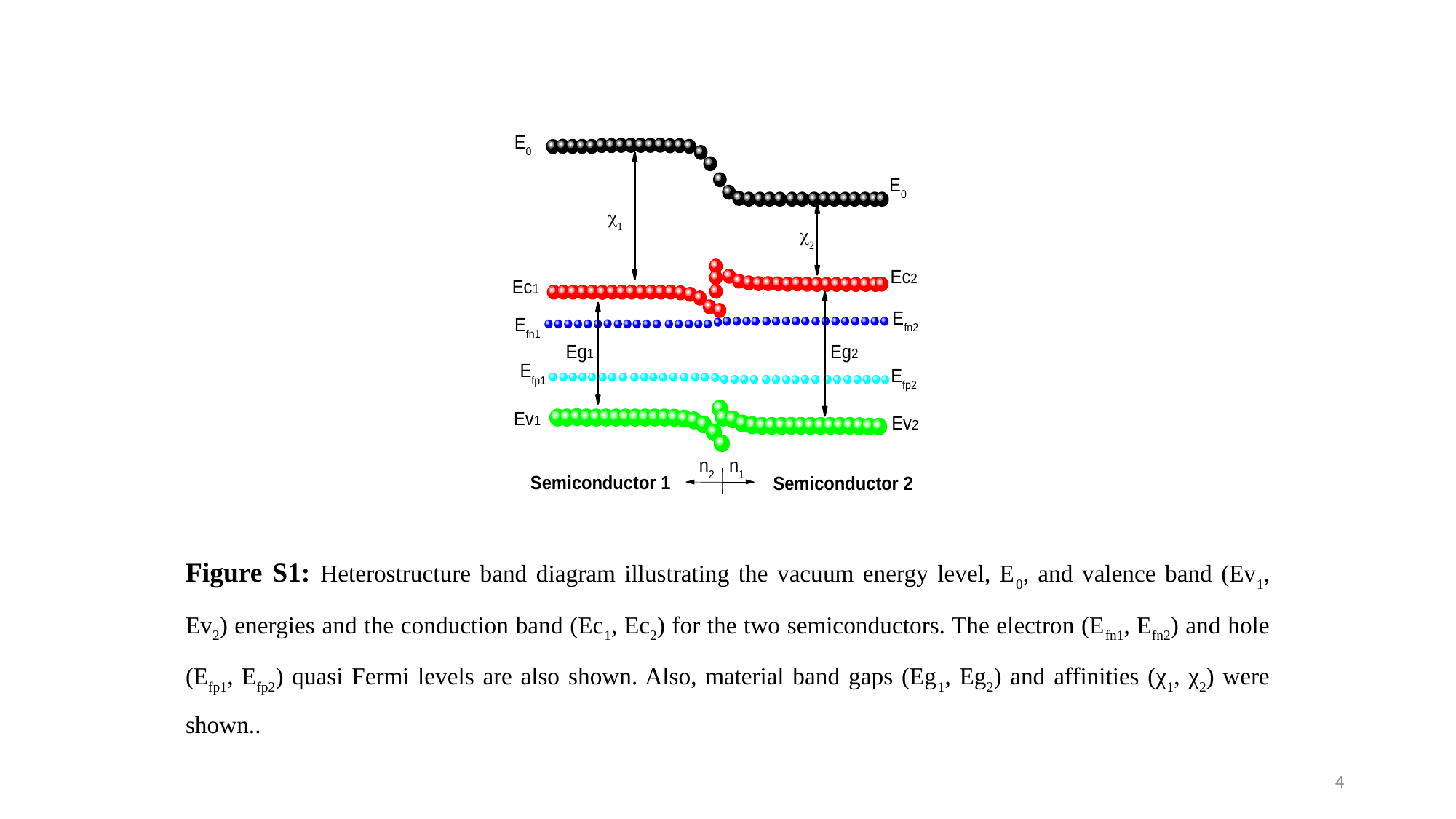

Figure S1: Heterostructure band diagram illustrating the vacuum energy level, E0, and valence band (Ev1, Ev2) energies and the conduction band (Ec1, Ec2) for the two semiconductors. The electron (Efn1, Efn2) and hole (Efp1, Efp2) quasi Fermi levels are also shown. Also, material band gaps (Eg1, Eg2) and affinities (χ1, χ2) were shown..
4

## Slide 5
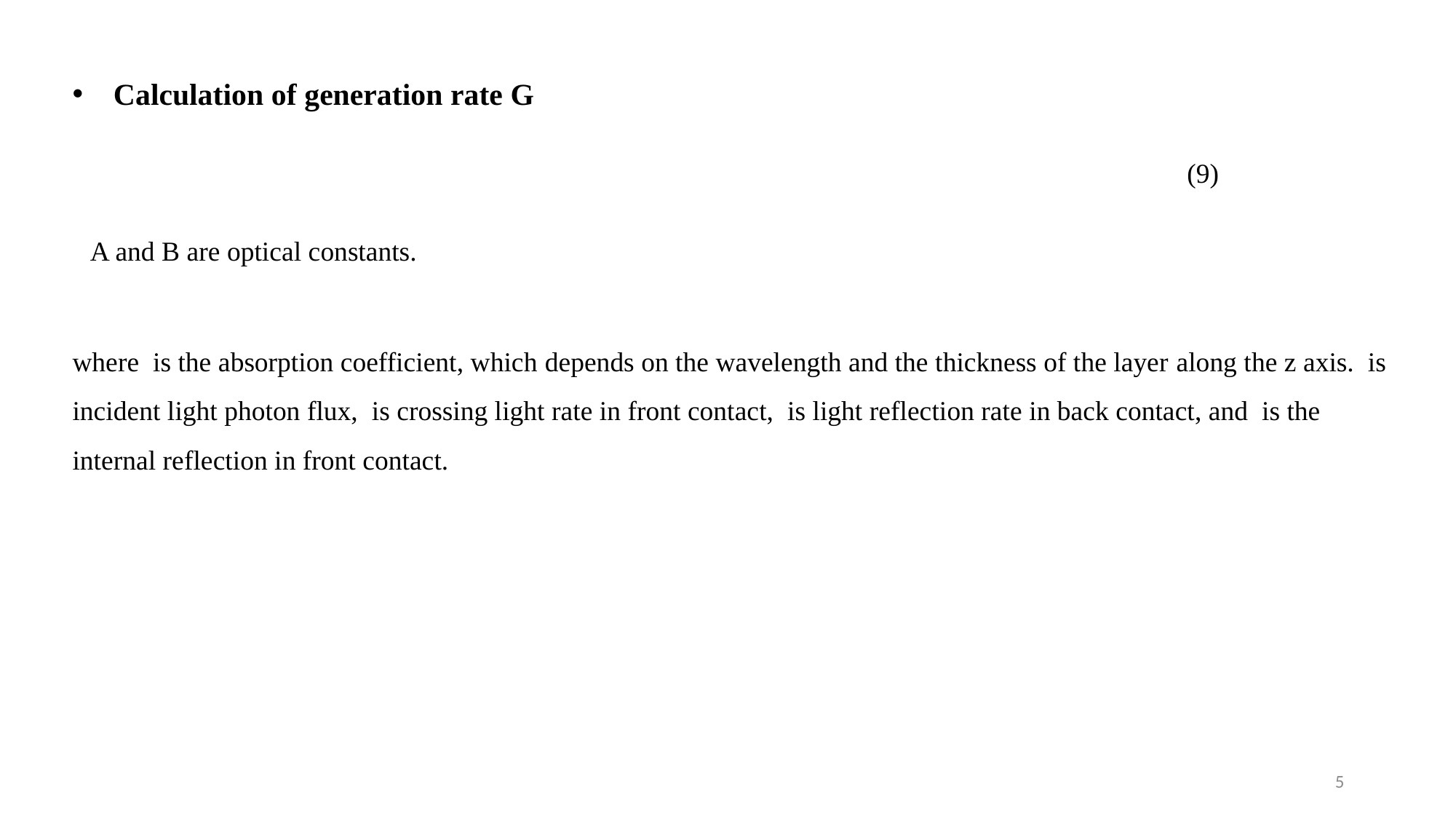

Calculation of generation rate G
A and B are optical constants.
5

## Slide 6
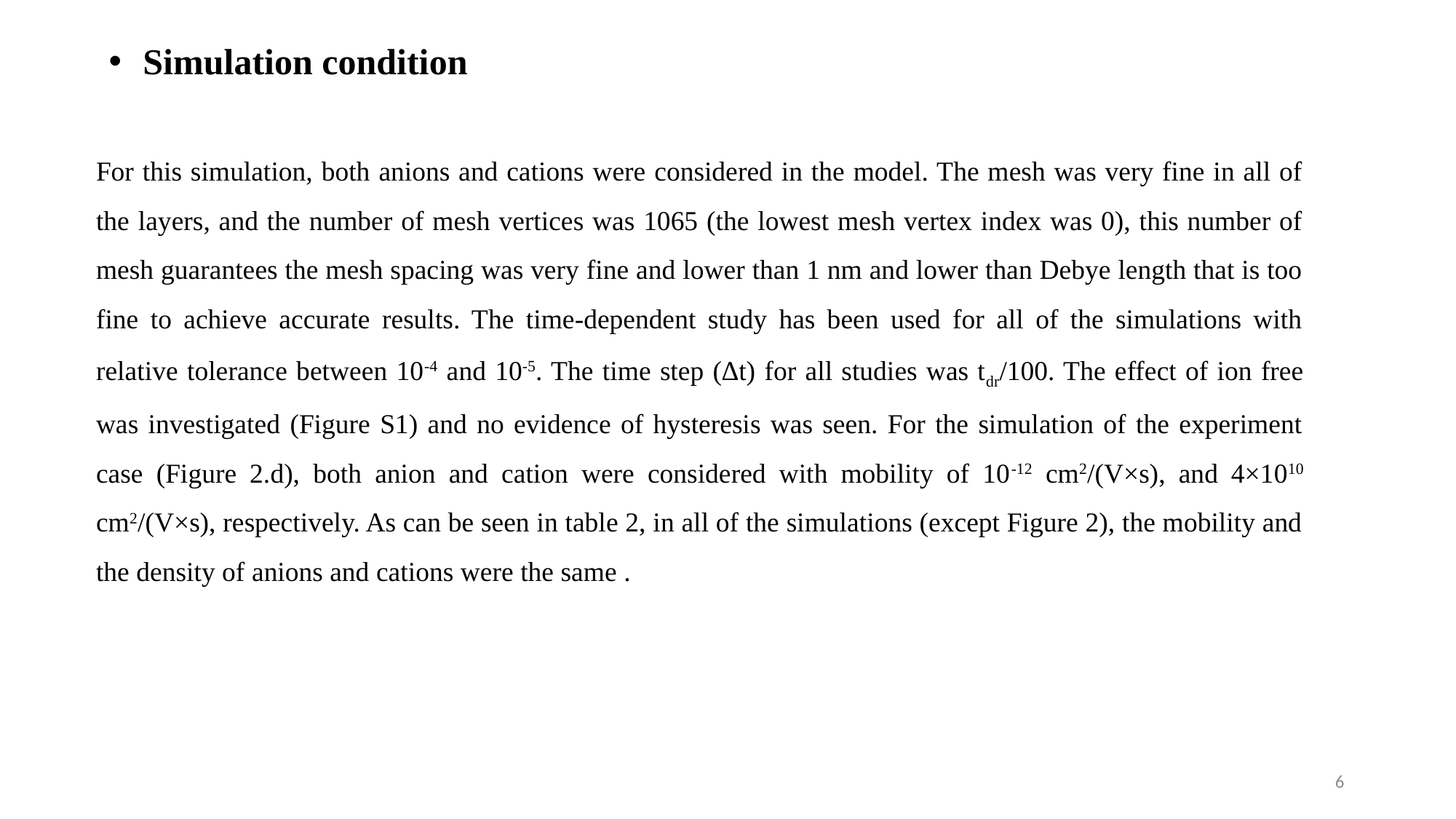

Simulation condition
For this simulation, both anions and cations were considered in the model. The mesh was very fine in all of the layers, and the number of mesh vertices was 1065 (the lowest mesh vertex index was 0), this number of mesh guarantees the mesh spacing was very fine and lower than 1 nm and lower than Debye length that is too fine to achieve accurate results. The time-dependent study has been used for all of the simulations with relative tolerance between 10-4 and 10-5. The time step (∆t) for all studies was tdr/100. The effect of ion free was investigated (Figure S1) and no evidence of hysteresis was seen. For the simulation of the experiment case (Figure 2.d), both anion and cation were considered with mobility of 10-12 cm2/(V×s), and 4×10^10^ cm2/(V×s), respectively. As can be seen in table 2, in all of the simulations (except Figure 2), the mobility and the density of anions and cations were the same .
6

## Slide 7
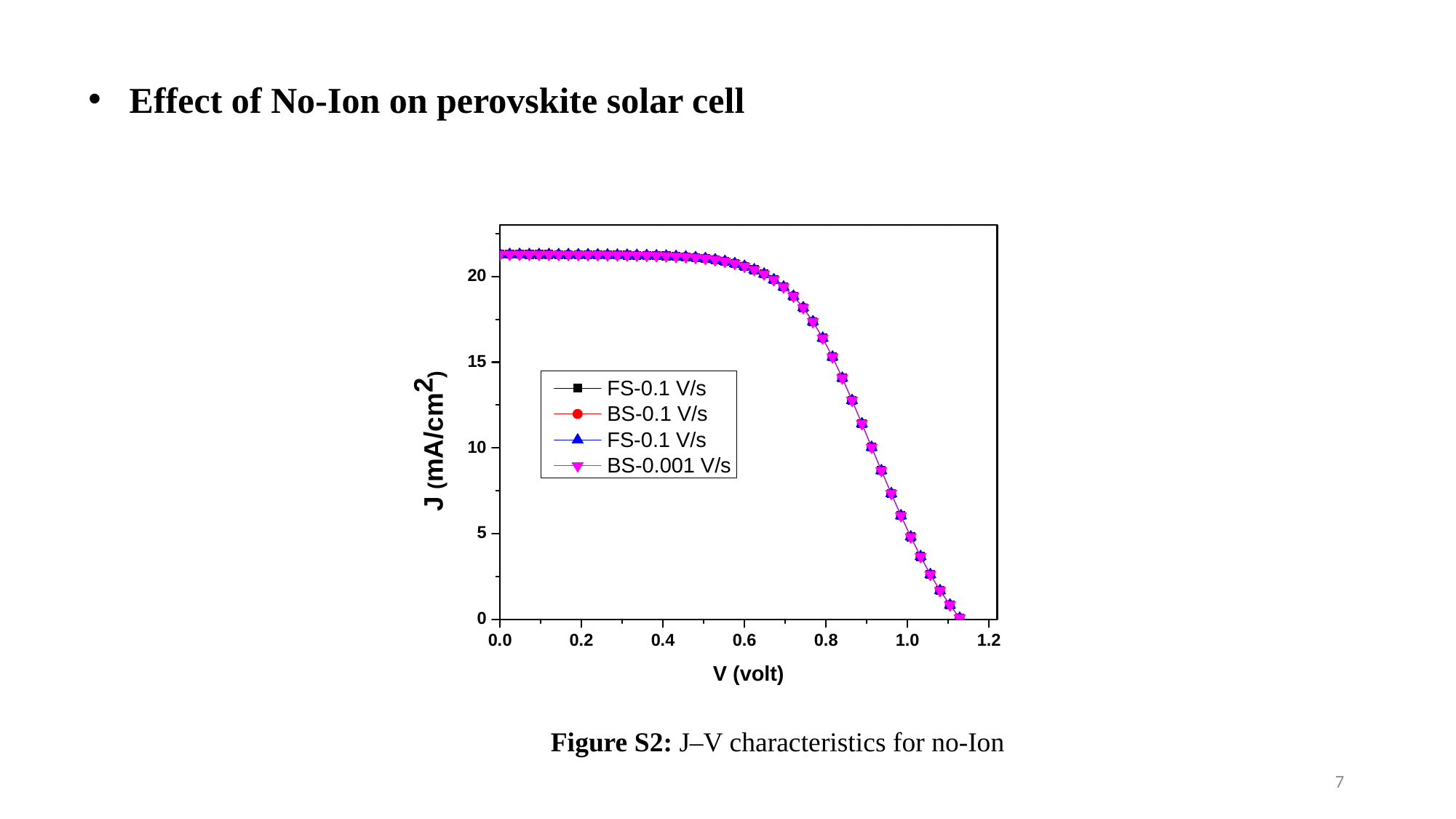

Effect of No-Ion on perovskite solar cell
Figure S2: J–V characteristics for no-Ion
7

## Slide 8
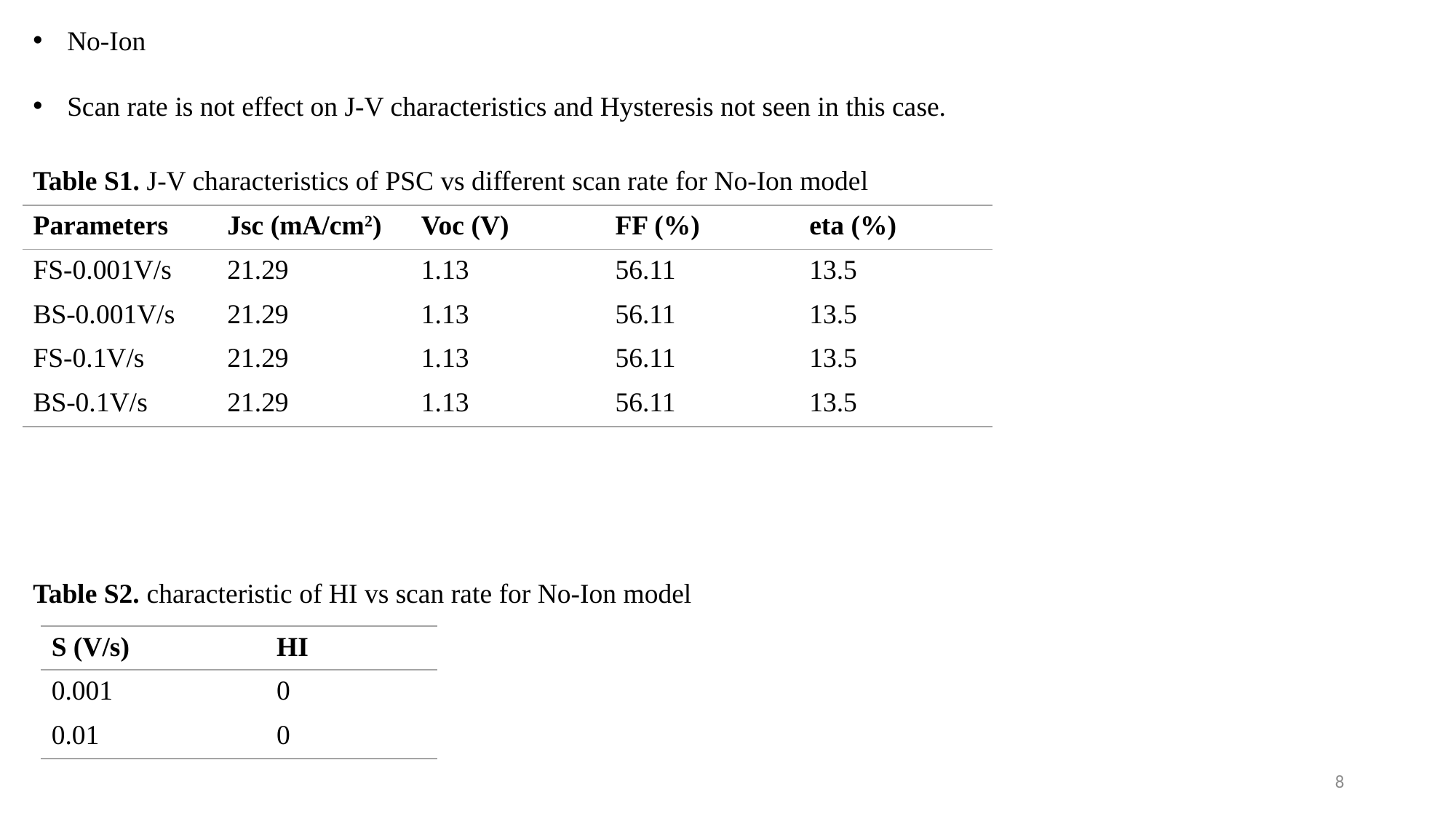

No-Ion
Scan rate is not effect on J-V characteristics and Hysteresis not seen in this case.
Table S1. J-V characteristics of PSC vs different scan rate for No-Ion model
| Parameters | Jsc (mA/cm2) | Voc (V) | FF (%) | eta (%) |
| --- | --- | --- | --- | --- |
| FS-0.001V/s | 21.29 | 1.13 | 56.11 | 13.5 |
| BS-0.001V/s | 21.29 | 1.13 | 56.11 | 13.5 |
| FS-0.1V/s | 21.29 | 1.13 | 56.11 | 13.5 |
| BS-0.1V/s | 21.29 | 1.13 | 56.11 | 13.5 |
Table S2. characteristic of HI vs scan rate for No-Ion model
| S (V/s) | HI |
| --- | --- |
| 0.001 | 0 |
| 0.01 | 0 |
8

## Slide 9
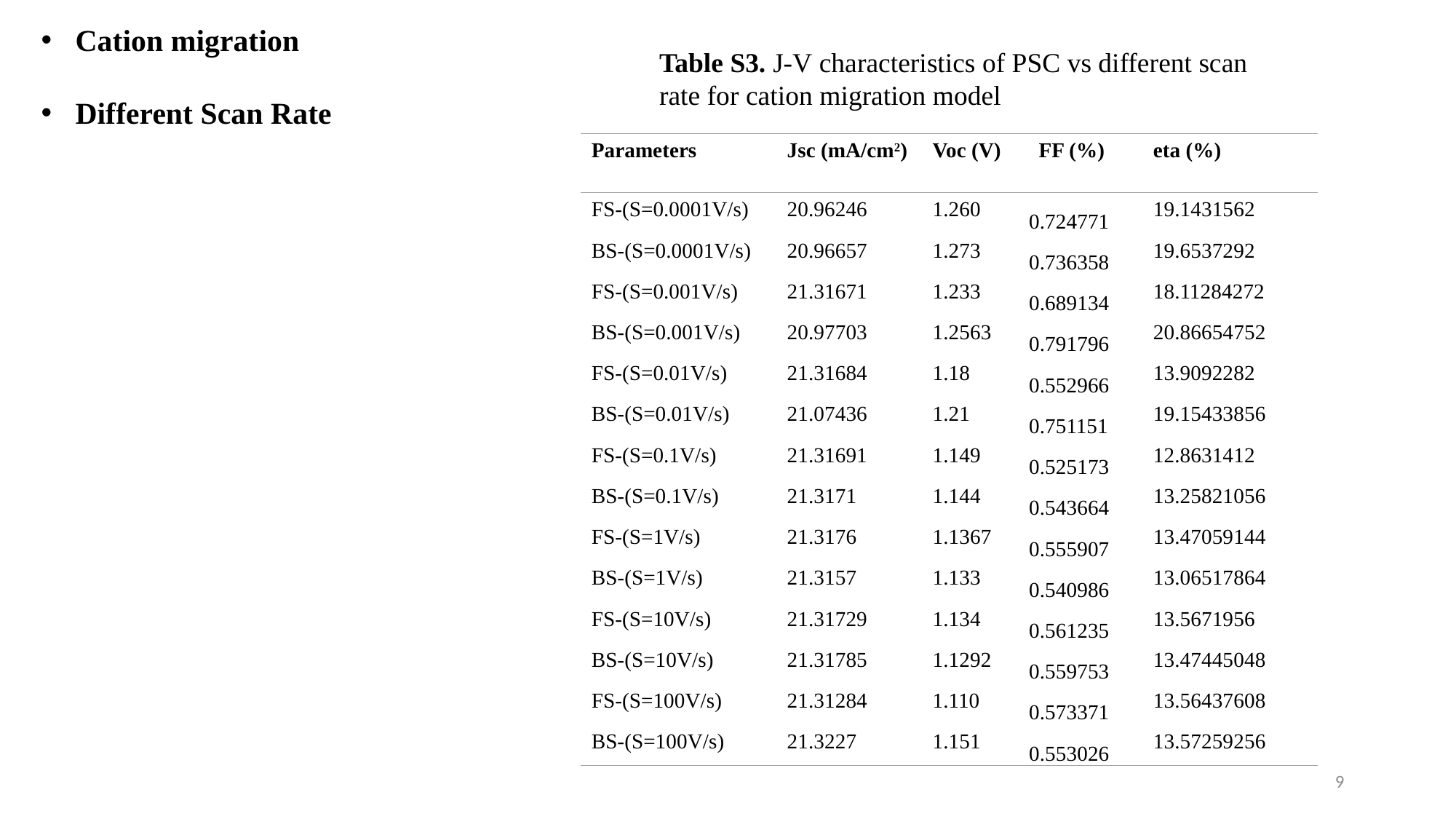

Cation migration
Different Scan Rate
Table S3. J-V characteristics of PSC vs different scan rate for cation migration model
| Parameters | Jsc (mA/cm2) | Voc (V) | FF (%) | eta (%) |
| --- | --- | --- | --- | --- |
| FS-(S=0.0001V/s) | 20.96246 | 1.260 | 0.724771 | 19.1431562 |
| BS-(S=0.0001V/s) | 20.96657 | 1.273 | 0.736358 | 19.6537292 |
| FS-(S=0.001V/s) | 21.31671 | 1.233 | 0.689134 | 18.11284272 |
| BS-(S=0.001V/s) | 20.97703 | 1.2563 | 0.791796 | 20.86654752 |
| FS-(S=0.01V/s) | 21.31684 | 1.18 | 0.552966 | 13.9092282 |
| BS-(S=0.01V/s) | 21.07436 | 1.21 | 0.751151 | 19.15433856 |
| FS-(S=0.1V/s) | 21.31691 | 1.149 | 0.525173 | 12.8631412 |
| BS-(S=0.1V/s) | 21.3171 | 1.144 | 0.543664 | 13.25821056 |
| FS-(S=1V/s) | 21.3176 | 1.1367 | 0.555907 | 13.47059144 |
| BS-(S=1V/s) | 21.3157 | 1.133 | 0.540986 | 13.06517864 |
| FS-(S=10V/s) | 21.31729 | 1.134 | 0.561235 | 13.5671956 |
| BS-(S=10V/s) | 21.31785 | 1.1292 | 0.559753 | 13.47445048 |
| FS-(S=100V/s) | 21.31284 | 1.110 | 0.573371 | 13.56437608 |
| BS-(S=100V/s) | 21.3227 | 1.151 | 0.553026 | 13.57259256 |
9

## Slide 10
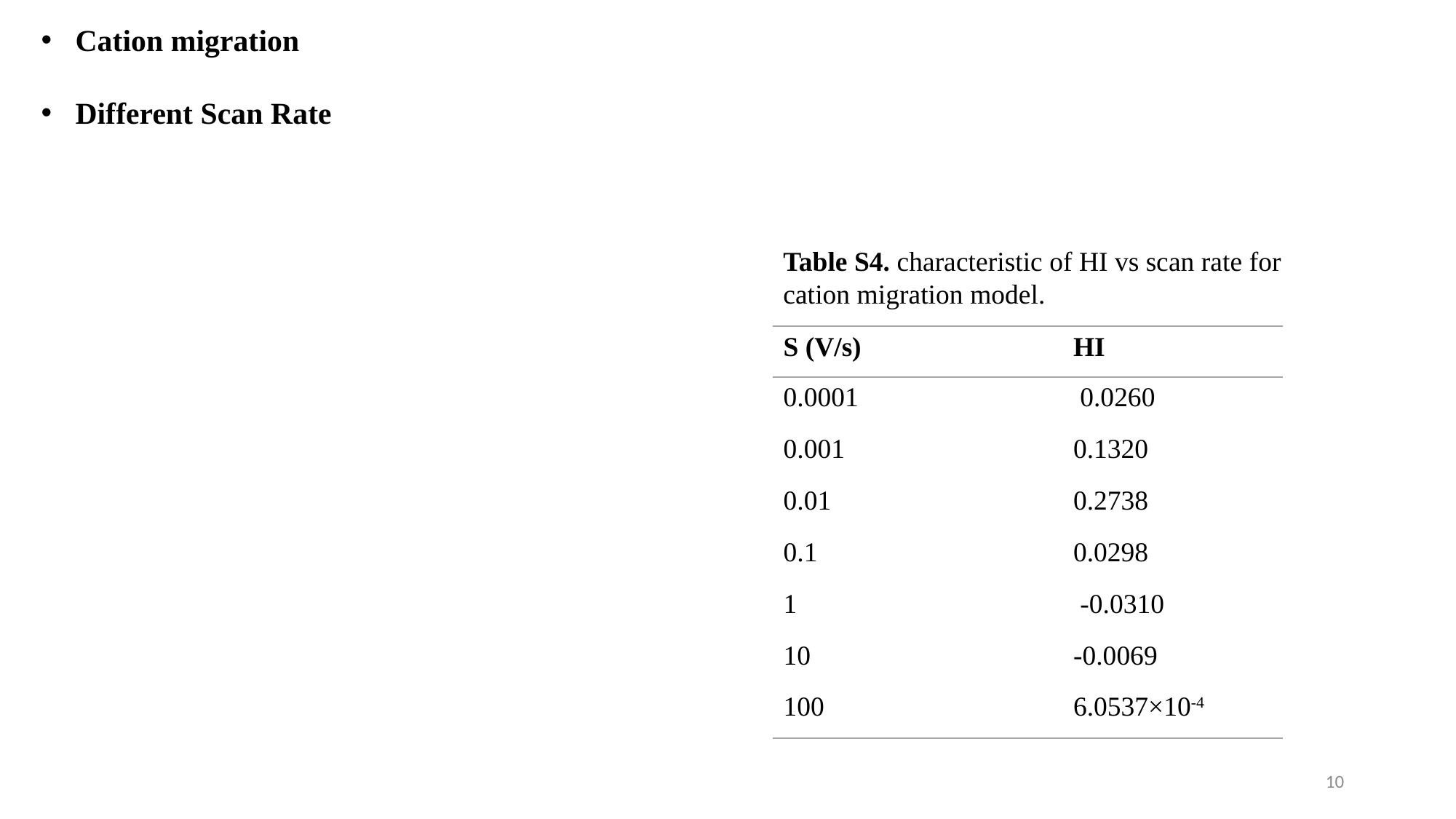

Cation migration
Different Scan Rate
Table S4. characteristic of HI vs scan rate for cation migration model.
| S (V/s) | HI |
| --- | --- |
| 0.0001 | 0.0260 |
| 0.001 | 0.1320 |
| 0.01 | 0.2738 |
| 0.1 | 0.0298 |
| 1 | -0.0310 |
| 10 | -0.0069 |
| 100 | 6.0537×10^-4^ |
10

## Slide 11
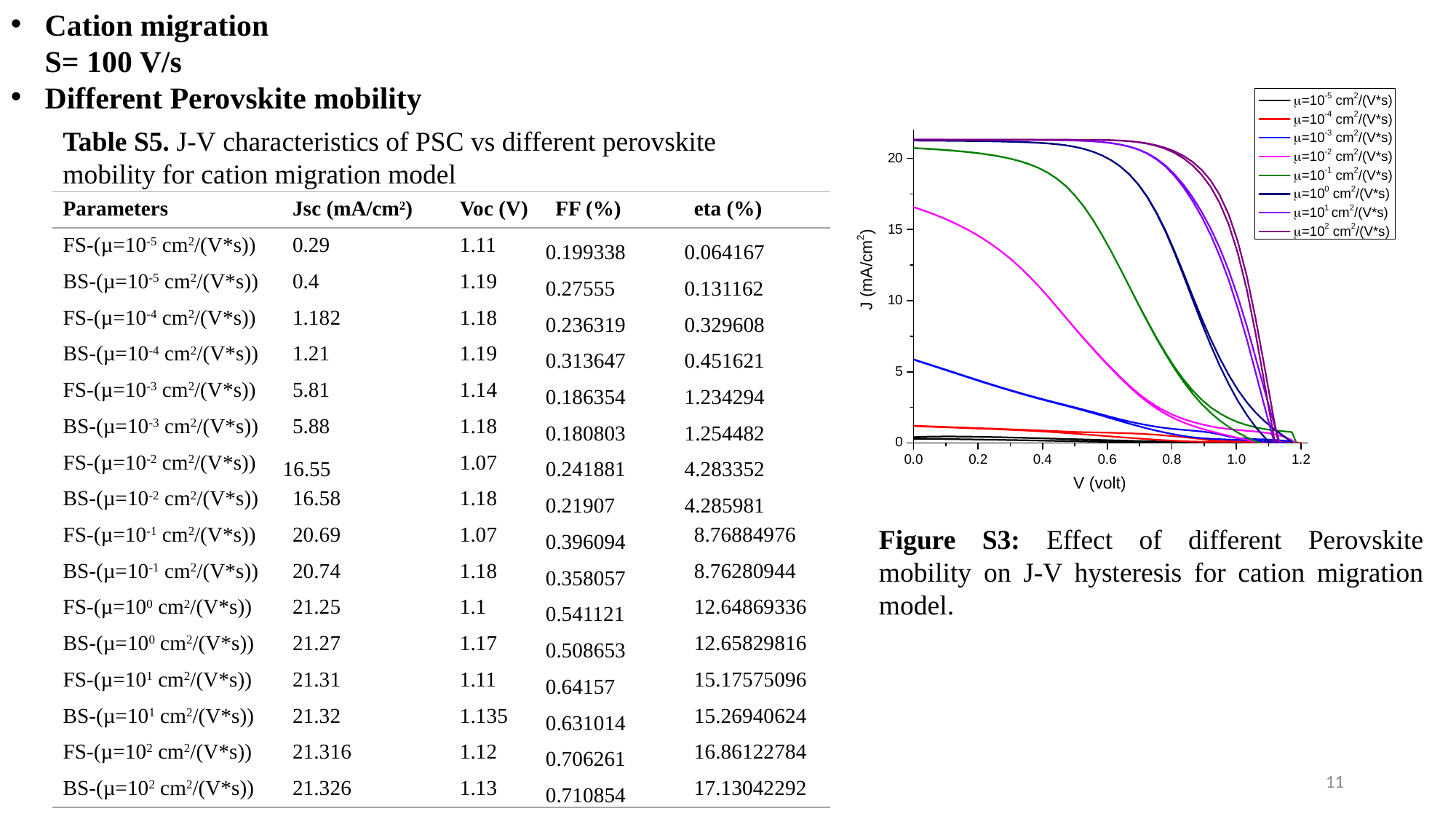

Cation migration
S= 100 V/s
Different Perovskite mobility
Table S5. J-V characteristics of PSC vs different perovskite mobility for cation migration model
| Parameters | Jsc (mA/cm2) | Voc (V) | FF (%) | eta (%) |
| --- | --- | --- | --- | --- |
| FS-(µ=10-5 cm2/(V\*s)) | 0.29 | 1.11 | 0.199338 | 0.064167 |
| BS-(µ=10-5 cm2/(V\*s)) | 0.4 | 1.19 | 0.27555 | 0.131162 |
| FS-(µ=10-4 cm2/(V\*s)) | 1.182 | 1.18 | 0.236319 | 0.329608 |
| BS-(µ=10-4 cm2/(V\*s)) | 1.21 | 1.19 | 0.313647 | 0.451621 |
| FS-(µ=10-3 cm2/(V\*s)) | 5.81 | 1.14 | 0.186354 | 1.234294 |
| BS-(µ=10-3 cm2/(V\*s)) | 5.88 | 1.18 | 0.180803 | 1.254482 |
| FS-(µ=10-2 cm2/(V\*s)) | 16.55 | 1.07 | 0.241881 | 4.283352 |
| BS-(µ=10-2 cm2/(V\*s)) | 16.58 | 1.18 | 0.21907 | 4.285981 |
| FS-(µ=10-1 cm2/(V\*s)) | 20.69 | 1.07 | 0.396094 | 8.76884976 |
| BS-(µ=10-1 cm2/(V\*s)) | 20.74 | 1.18 | 0.358057 | 8.76280944 |
| FS-(µ=100 cm2/(V\*s)) | 21.25 | 1.1 | 0.541121 | 12.64869336 |
| BS-(µ=100 cm2/(V\*s)) | 21.27 | 1.17 | 0.508653 | 12.65829816 |
| FS-(µ=101 cm2/(V\*s)) | 21.31 | 1.11 | 0.64157 | 15.17575096 |
| BS-(µ=101 cm2/(V\*s)) | 21.32 | 1.135 | 0.631014 | 15.26940624 |
| FS-(µ=102 cm2/(V\*s)) | 21.316 | 1.12 | 0.706261 | 16.86122784 |
| BS-(µ=102 cm2/(V\*s)) | 21.326 | 1.13 | 0.710854 | 17.13042292 |
Figure S3: Effect of different Perovskite mobility on J-V hysteresis for cation migration model.
11

## Slide 12
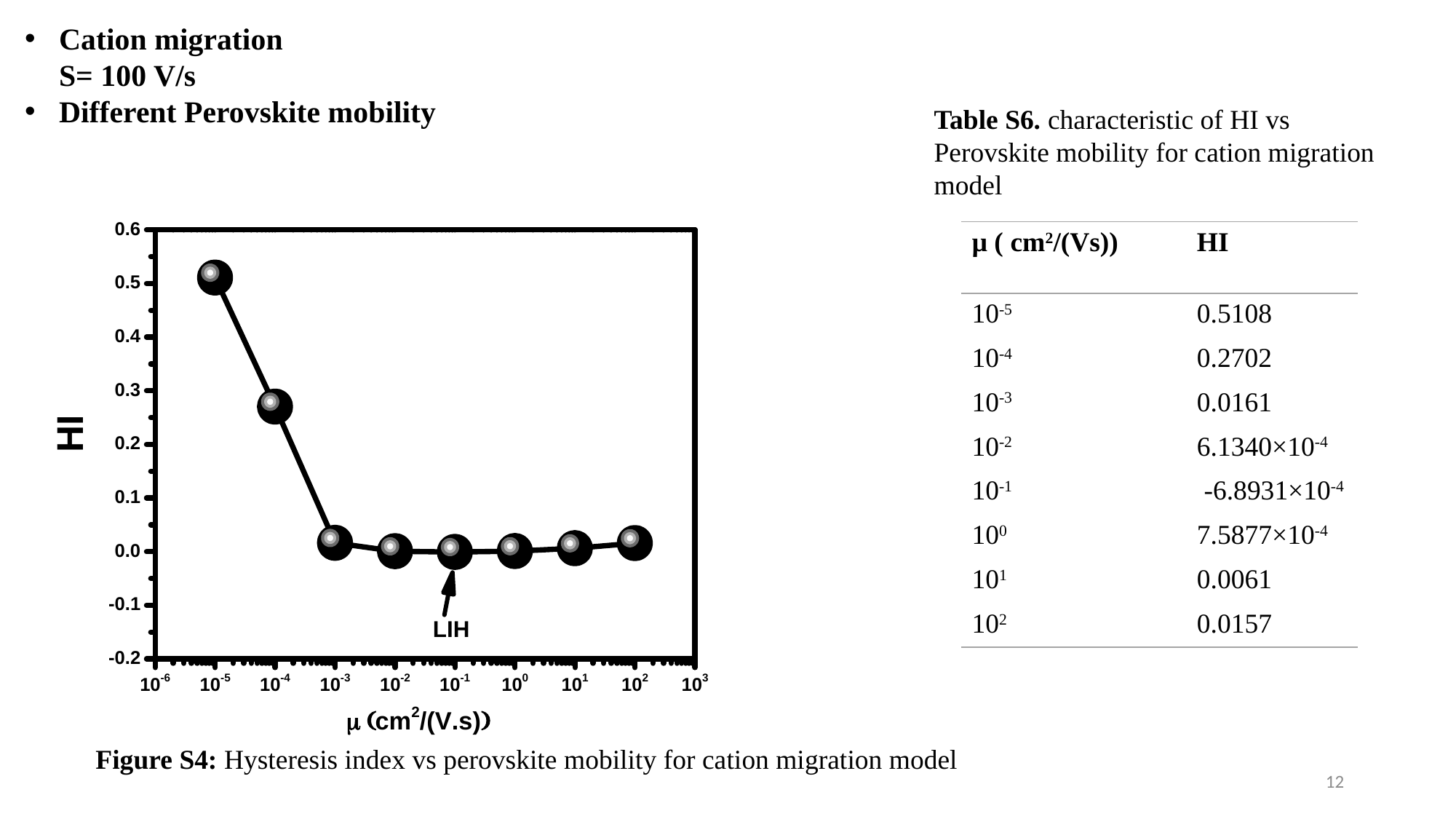

Cation migration
S= 100 V/s
Different Perovskite mobility
Table S6. characteristic of HI vs Perovskite mobility for cation migration model
Figure S4: Hysteresis index vs perovskite mobility for cation migration model
12

## Slide 13
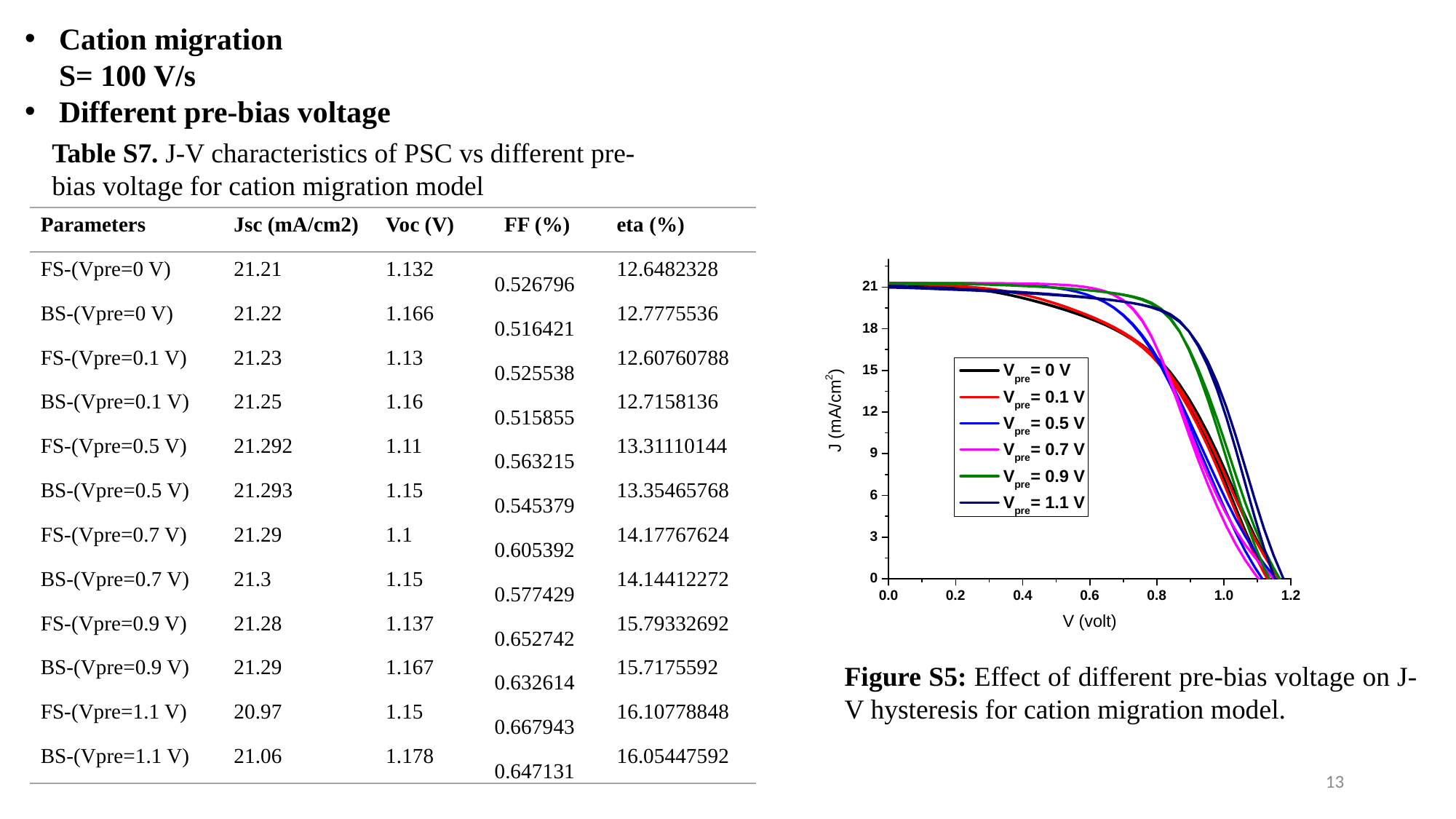

Cation migration
S= 100 V/s
Different pre-bias voltage
Table S7. J-V characteristics of PSC vs different pre-bias voltage for cation migration model
| Parameters | Jsc (mA/cm2) | Voc (V) | FF (%) | eta (%) |
| --- | --- | --- | --- | --- |
| FS-(Vpre=0 V) | 21.21 | 1.132 | 0.526796 | 12.6482328 |
| BS-(Vpre=0 V) | 21.22 | 1.166 | 0.516421 | 12.7775536 |
| FS-(Vpre=0.1 V) | 21.23 | 1.13 | 0.525538 | 12.60760788 |
| BS-(Vpre=0.1 V) | 21.25 | 1.16 | 0.515855 | 12.7158136 |
| FS-(Vpre=0.5 V) | 21.292 | 1.11 | 0.563215 | 13.31110144 |
| BS-(Vpre=0.5 V) | 21.293 | 1.15 | 0.545379 | 13.35465768 |
| FS-(Vpre=0.7 V) | 21.29 | 1.1 | 0.605392 | 14.17767624 |
| BS-(Vpre=0.7 V) | 21.3 | 1.15 | 0.577429 | 14.14412272 |
| FS-(Vpre=0.9 V) | 21.28 | 1.137 | 0.652742 | 15.79332692 |
| BS-(Vpre=0.9 V) | 21.29 | 1.167 | 0.632614 | 15.7175592 |
| FS-(Vpre=1.1 V) | 20.97 | 1.15 | 0.667943 | 16.10778848 |
| BS-(Vpre=1.1 V) | 21.06 | 1.178 | 0.647131 | 16.05447592 |
Figure S5: Effect of different pre-bias voltage on J-V hysteresis for cation migration model.
13

## Slide 14
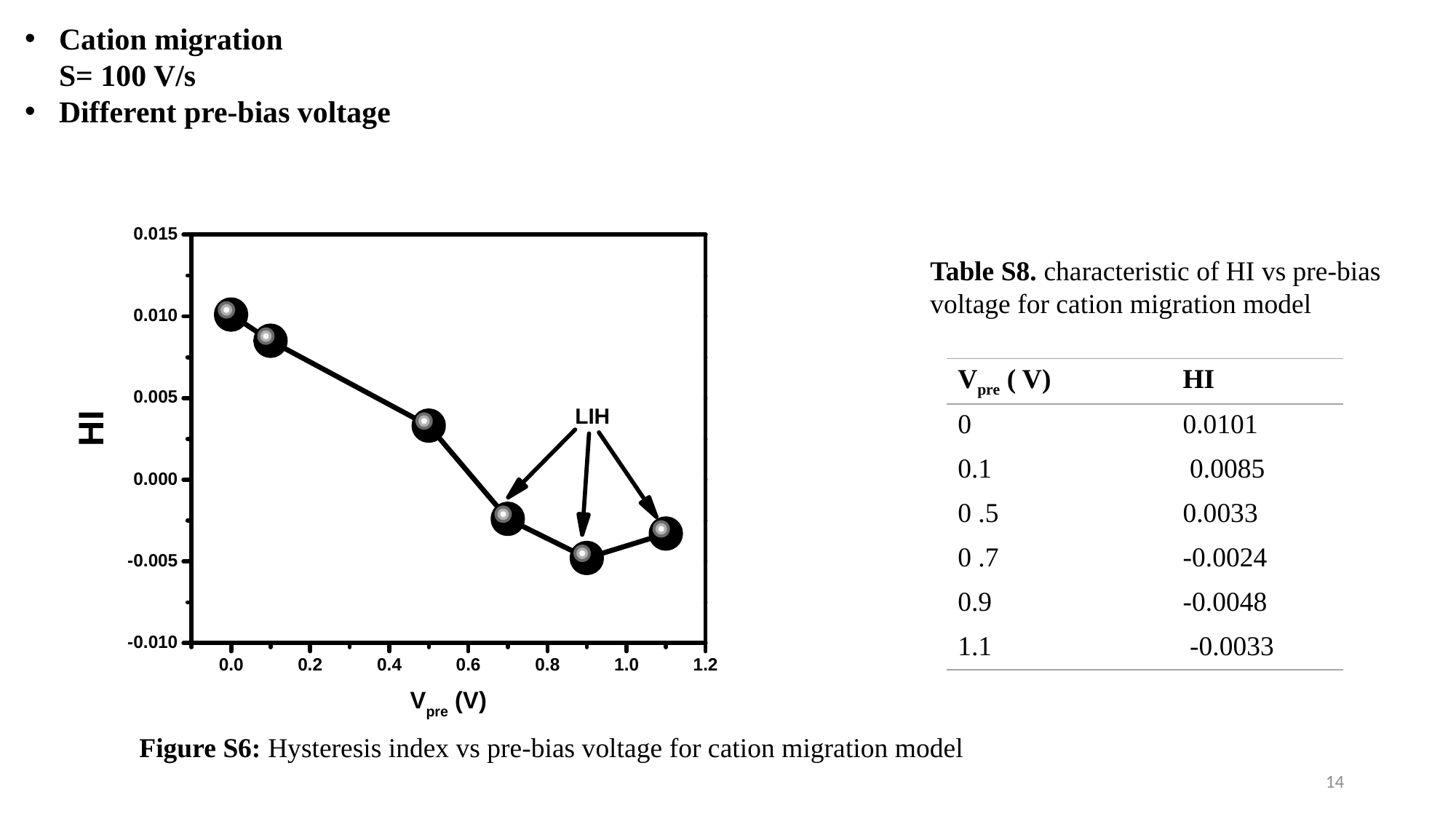

Cation migration
S= 100 V/s
Different pre-bias voltage
Table S8. characteristic of HI vs pre-bias voltage for cation migration model
| Vpre ( V) | HI |
| --- | --- |
| 0 | 0.0101 |
| 0.1 | 0.0085 |
| 0 .5 | 0.0033 |
| 0 .7 | -0.0024 |
| 0.9 | -0.0048 |
| 1.1 | -0.0033 |
Figure S6: Hysteresis index vs pre-bias voltage for cation migration model
14

## Slide 15
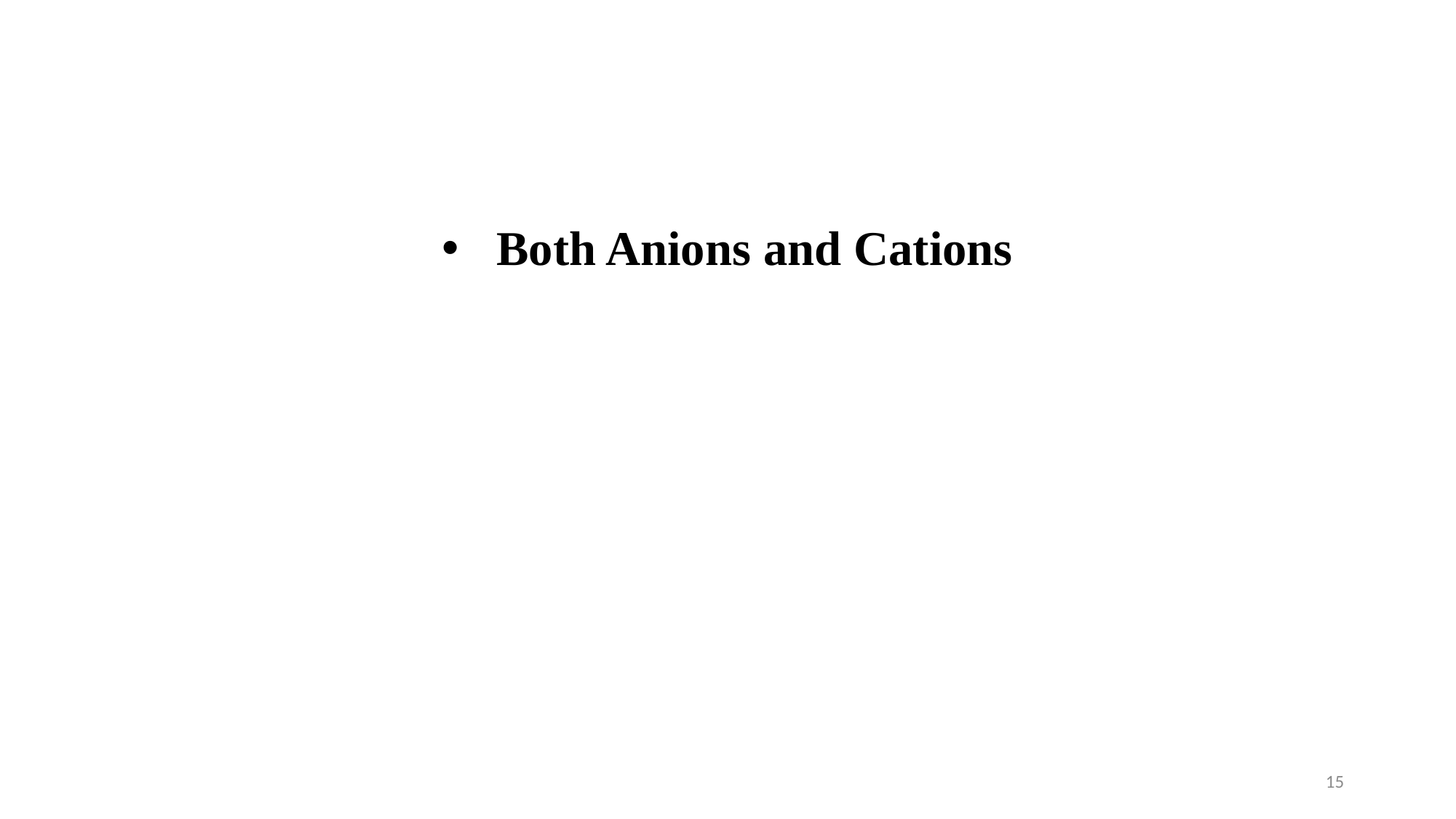

Both Anions and Cations
15

## Slide 16
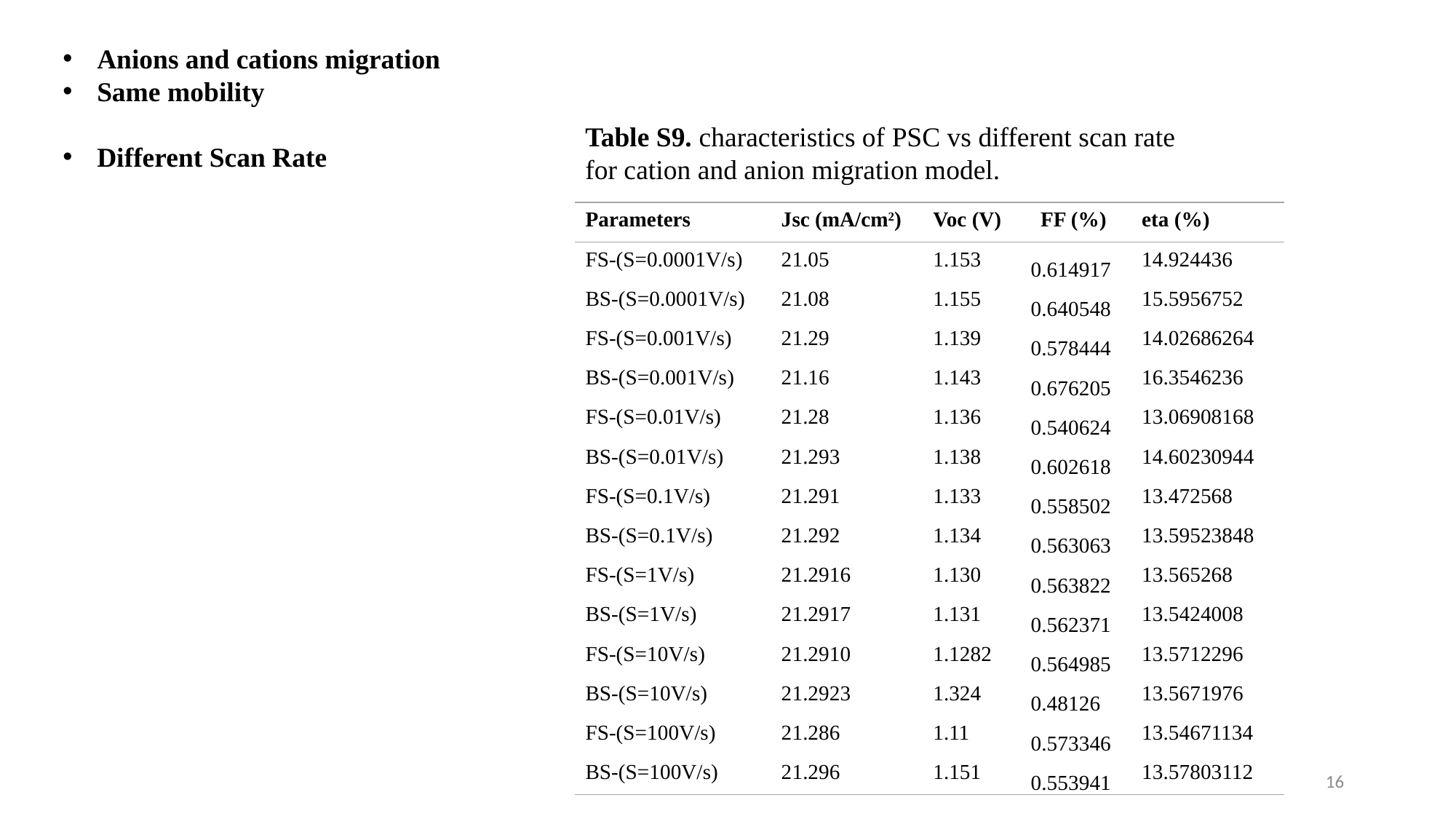

Anions and cations migration
Same mobility
Different Scan Rate
Table S9. characteristics of PSC vs different scan rate for cation and anion migration model.
| Parameters | Jsc (mA/cm2) | Voc (V) | FF (%) | eta (%) |
| --- | --- | --- | --- | --- |
| FS-(S=0.0001V/s) | 21.05 | 1.153 | 0.614917 | 14.924436 |
| BS-(S=0.0001V/s) | 21.08 | 1.155 | 0.640548 | 15.5956752 |
| FS-(S=0.001V/s) | 21.29 | 1.139 | 0.578444 | 14.02686264 |
| BS-(S=0.001V/s) | 21.16 | 1.143 | 0.676205 | 16.3546236 |
| FS-(S=0.01V/s) | 21.28 | 1.136 | 0.540624 | 13.06908168 |
| BS-(S=0.01V/s) | 21.293 | 1.138 | 0.602618 | 14.60230944 |
| FS-(S=0.1V/s) | 21.291 | 1.133 | 0.558502 | 13.472568 |
| BS-(S=0.1V/s) | 21.292 | 1.134 | 0.563063 | 13.59523848 |
| FS-(S=1V/s) | 21.2916 | 1.130 | 0.563822 | 13.565268 |
| BS-(S=1V/s) | 21.2917 | 1.131 | 0.562371 | 13.5424008 |
| FS-(S=10V/s) | 21.2910 | 1.1282 | 0.564985 | 13.5712296 |
| BS-(S=10V/s) | 21.2923 | 1.324 | 0.48126 | 13.5671976 |
| FS-(S=100V/s) | 21.286 | 1.11 | 0.573346 | 13.54671134 |
| BS-(S=100V/s) | 21.296 | 1.151 | 0.553941 | 13.57803112 |
16

## Slide 17
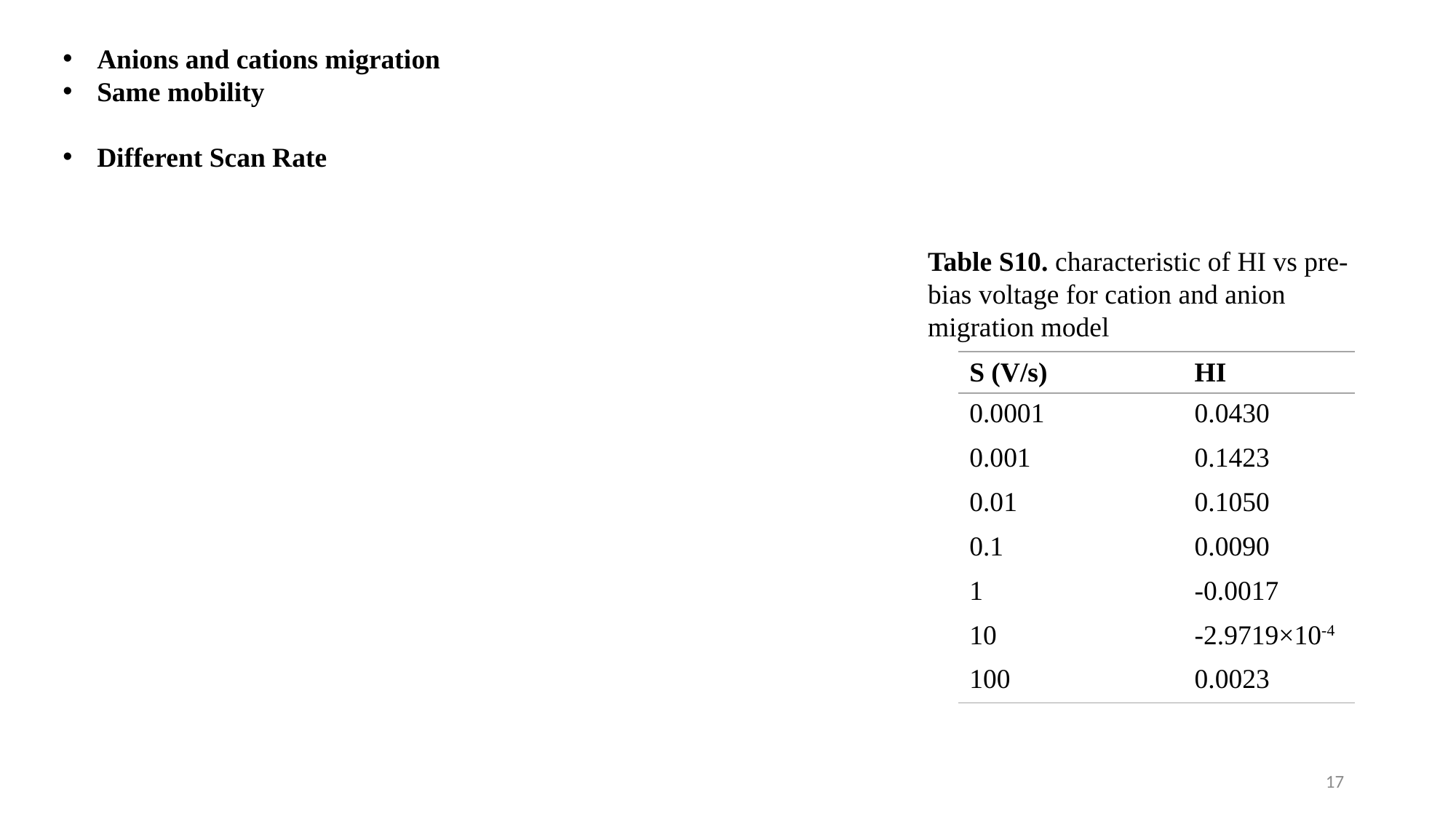

Anions and cations migration
Same mobility
Different Scan Rate
Table S10. characteristic of HI vs pre-bias voltage for cation and anion migration model
| S (V/s) | HI |
| --- | --- |
| 0.0001 | 0.0430 |
| 0.001 | 0.1423 |
| 0.01 | 0.1050 |
| 0.1 | 0.0090 |
| 1 | -0.0017 |
| 10 | -2.9719×10^-4^ |
| 100 | 0.0023 |
17

## Slide 18
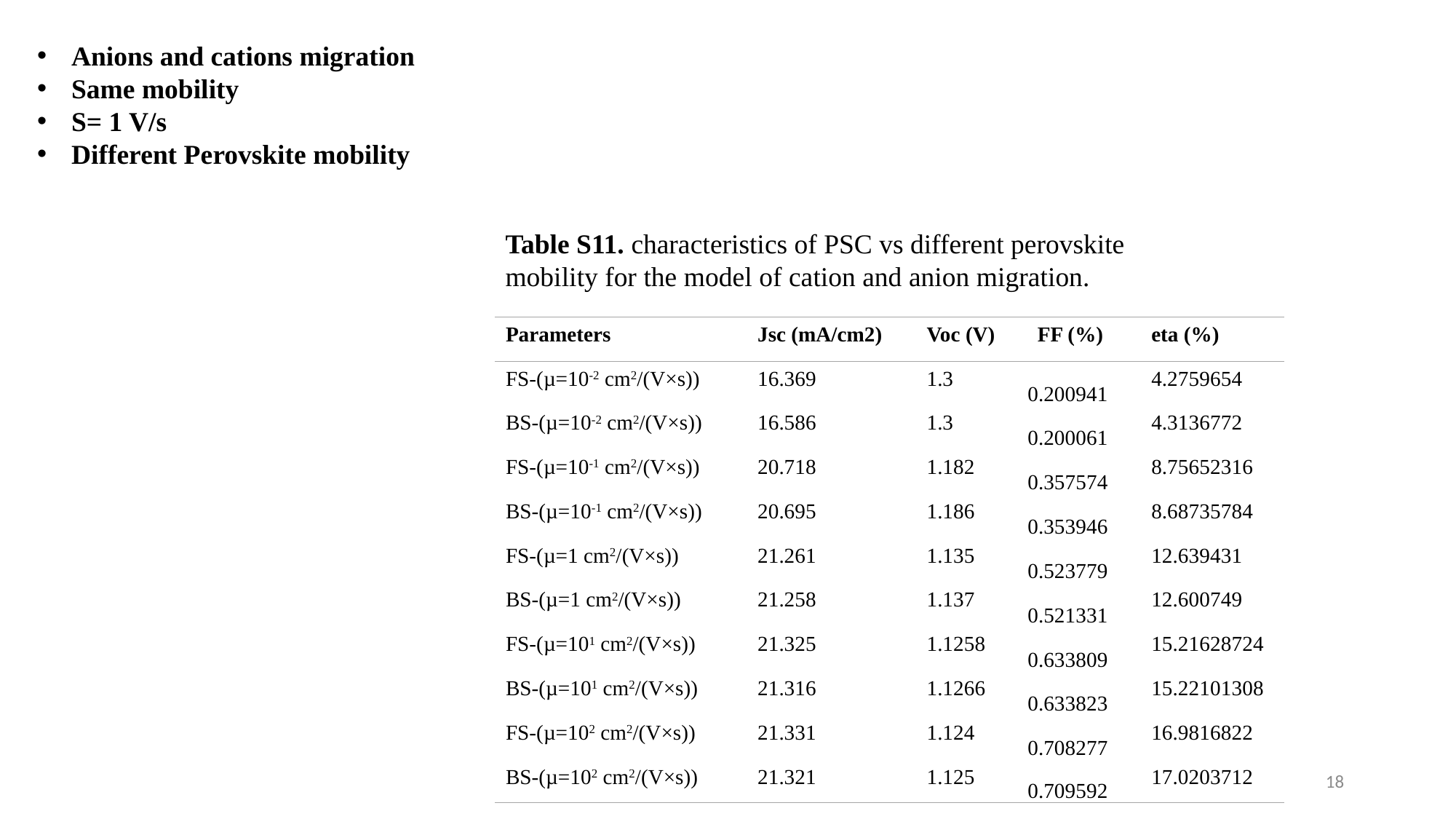

Anions and cations migration
Same mobility
S= 1 V/s
Different Perovskite mobility
Table S11. characteristics of PSC vs different perovskite mobility for the model of cation and anion migration.
| Parameters | Jsc (mA/cm2) | Voc (V) | FF (%) | eta (%) |
| --- | --- | --- | --- | --- |
| FS-(µ=10-2 cm2/(V×s)) | 16.369 | 1.3 | 0.200941 | 4.2759654 |
| BS-(µ=10-2 cm2/(V×s)) | 16.586 | 1.3 | 0.200061 | 4.3136772 |
| FS-(µ=10-1 cm2/(V×s)) | 20.718 | 1.182 | 0.357574 | 8.75652316 |
| BS-(µ=10-1 cm2/(V×s)) | 20.695 | 1.186 | 0.353946 | 8.68735784 |
| FS-(µ=1 cm2/(V×s)) | 21.261 | 1.135 | 0.523779 | 12.639431 |
| BS-(µ=1 cm2/(V×s)) | 21.258 | 1.137 | 0.521331 | 12.600749 |
| FS-(µ=101 cm2/(V×s)) | 21.325 | 1.1258 | 0.633809 | 15.21628724 |
| BS-(µ=101 cm2/(V×s)) | 21.316 | 1.1266 | 0.633823 | 15.22101308 |
| FS-(µ=102 cm2/(V×s)) | 21.331 | 1.124 | 0.708277 | 16.9816822 |
| BS-(µ=102 cm2/(V×s)) | 21.321 | 1.125 | 0.709592 | 17.0203712 |
18

## Slide 19
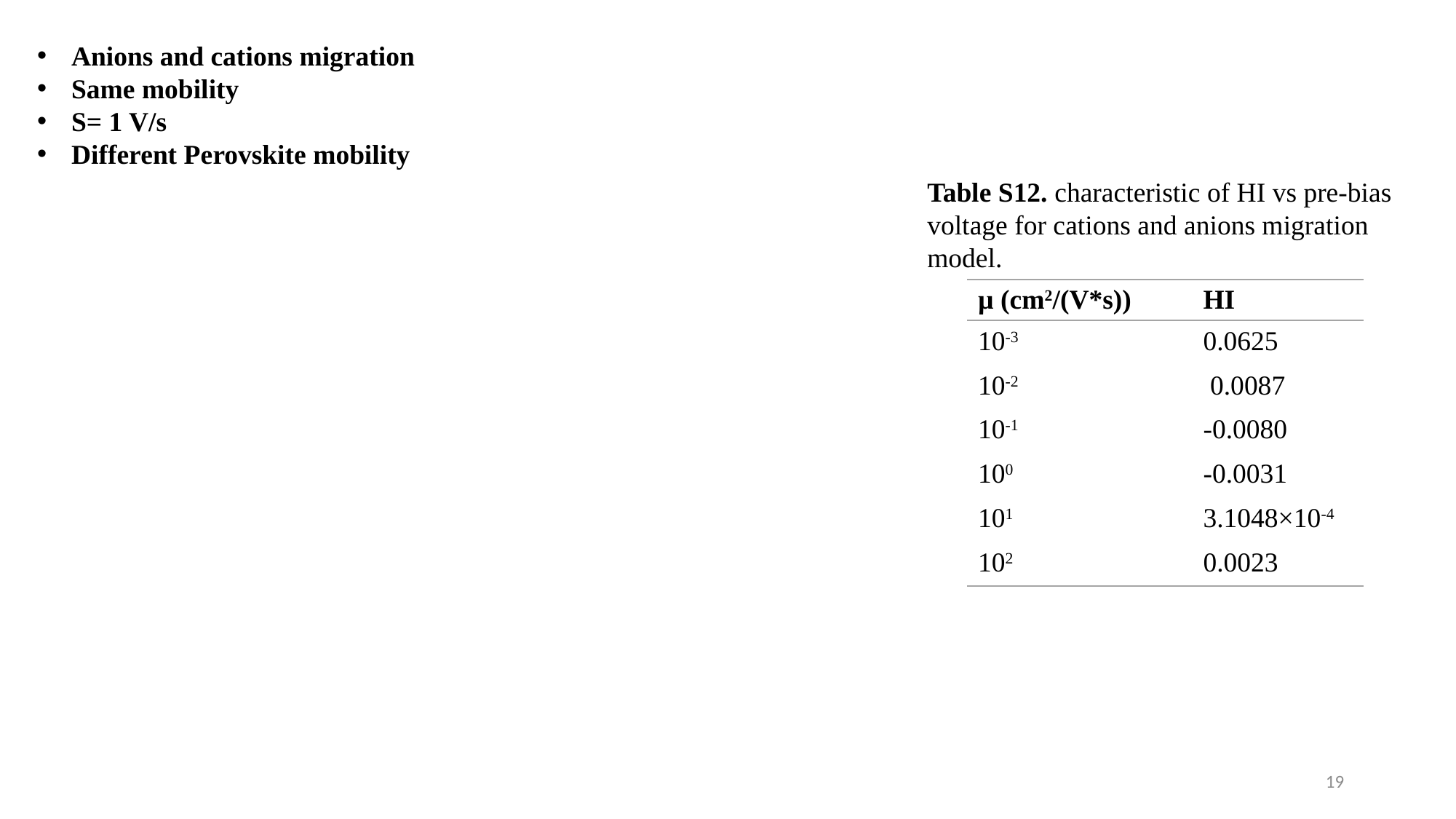

Anions and cations migration
Same mobility
S= 1 V/s
Different Perovskite mobility
Table S12. characteristic of HI vs pre-bias voltage for cations and anions migration model.
| µ (cm2/(V\*s)) | HI |
| --- | --- |
| 10-3 | 0.0625 |
| 10-2 | 0.0087 |
| 10-1 | -0.0080 |
| 100 | -0.0031 |
| 101 | 3.1048×10^-4^ |
| 102 | 0.0023 |
19

## Slide 20
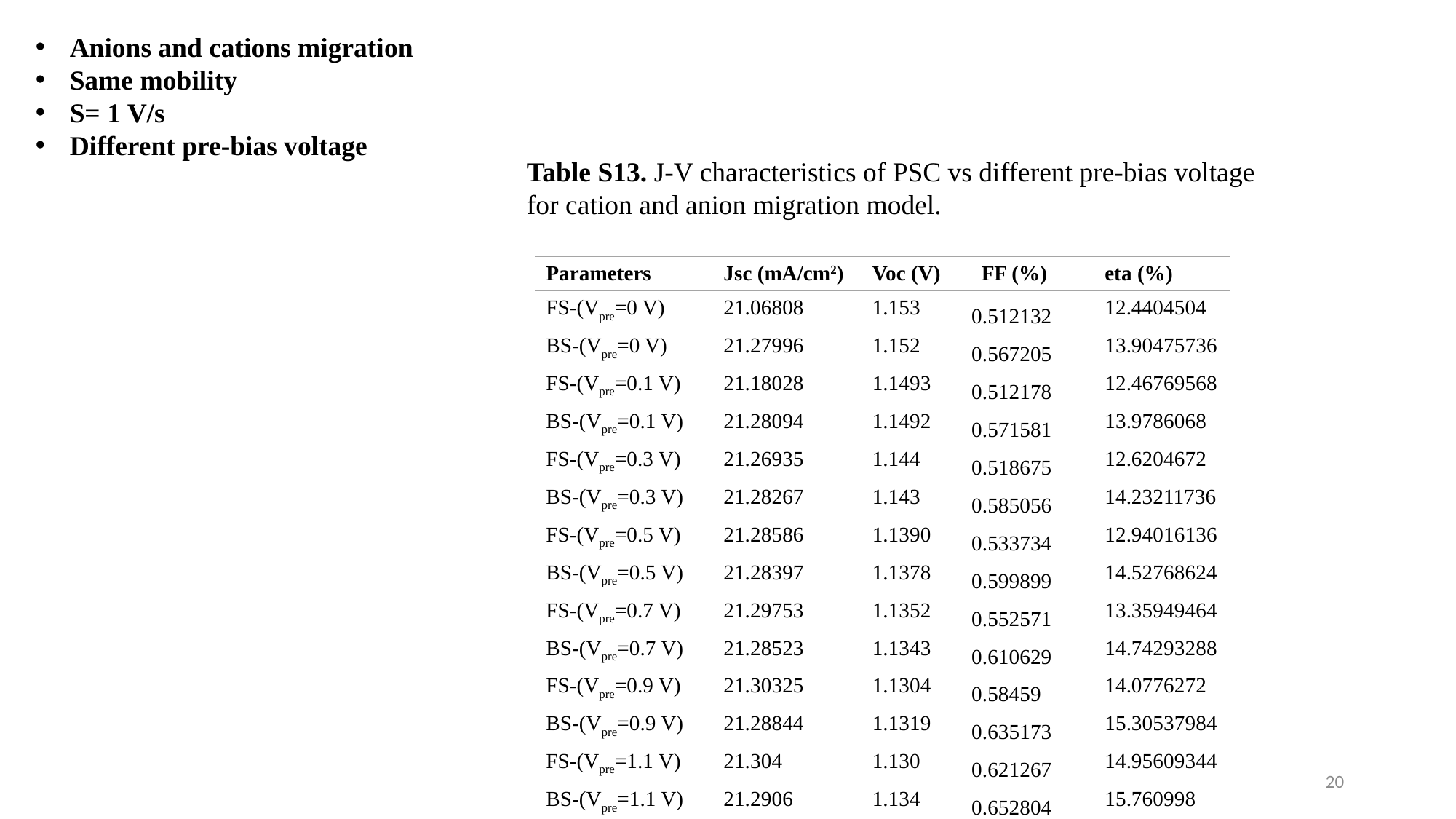

Anions and cations migration
Same mobility
S= 1 V/s
Different pre-bias voltage
Table S13. J-V characteristics of PSC vs different pre-bias voltage for cation and anion migration model.
| Parameters | Jsc (mA/cm2) | Voc (V) | FF (%) | eta (%) |
| --- | --- | --- | --- | --- |
| FS-(Vpre=0 V) | 21.06808 | 1.153 | 0.512132 | 12.4404504 |
| BS-(Vpre=0 V) | 21.27996 | 1.152 | 0.567205 | 13.90475736 |
| FS-(Vpre=0.1 V) | 21.18028 | 1.1493 | 0.512178 | 12.46769568 |
| BS-(Vpre=0.1 V) | 21.28094 | 1.1492 | 0.571581 | 13.9786068 |
| FS-(Vpre=0.3 V) | 21.26935 | 1.144 | 0.518675 | 12.6204672 |
| BS-(Vpre=0.3 V) | 21.28267 | 1.143 | 0.585056 | 14.23211736 |
| FS-(Vpre=0.5 V) | 21.28586 | 1.1390 | 0.533734 | 12.94016136 |
| BS-(Vpre=0.5 V) | 21.28397 | 1.1378 | 0.599899 | 14.52768624 |
| FS-(Vpre=0.7 V) | 21.29753 | 1.1352 | 0.552571 | 13.35949464 |
| BS-(Vpre=0.7 V) | 21.28523 | 1.1343 | 0.610629 | 14.74293288 |
| FS-(Vpre=0.9 V) | 21.30325 | 1.1304 | 0.58459 | 14.0776272 |
| BS-(Vpre=0.9 V) | 21.28844 | 1.1319 | 0.635173 | 15.30537984 |
| FS-(Vpre=1.1 V) | 21.304 | 1.130 | 0.621267 | 14.95609344 |
| BS-(Vpre=1.1 V) | 21.2906 | 1.134 | 0.652804 | 15.760998 |
20

## Slide 21
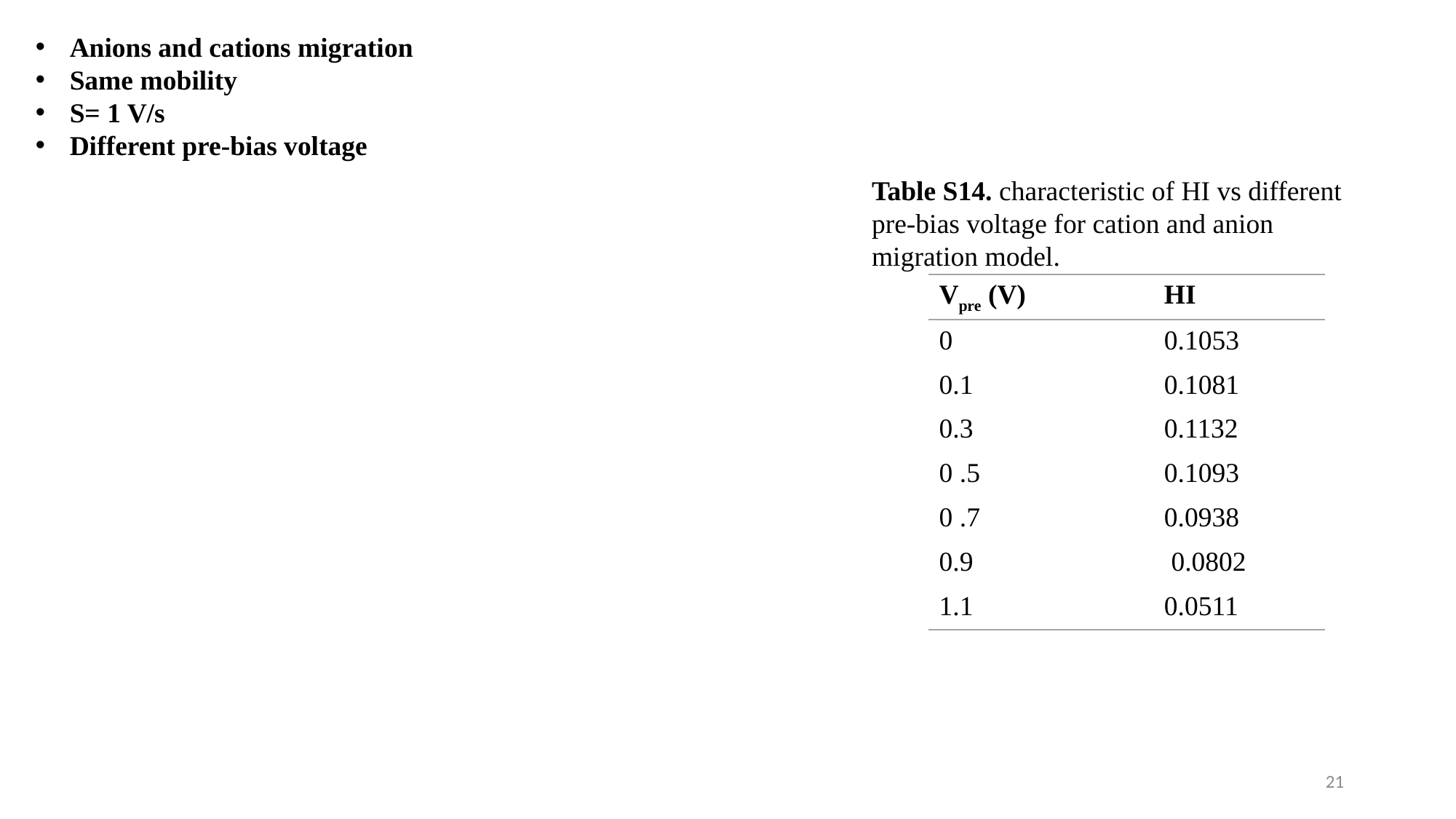

Anions and cations migration
Same mobility
S= 1 V/s
Different pre-bias voltage
Table S14. characteristic of HI vs different pre-bias voltage for cation and anion migration model.
| Vpre (V) | HI |
| --- | --- |
| 0 | 0.1053 |
| 0.1 | 0.1081 |
| 0.3 | 0.1132 |
| 0 .5 | 0.1093 |
| 0 .7 | 0.0938 |
| 0.9 | 0.0802 |
| 1.1 | 0.0511 |
21

## Slide 22
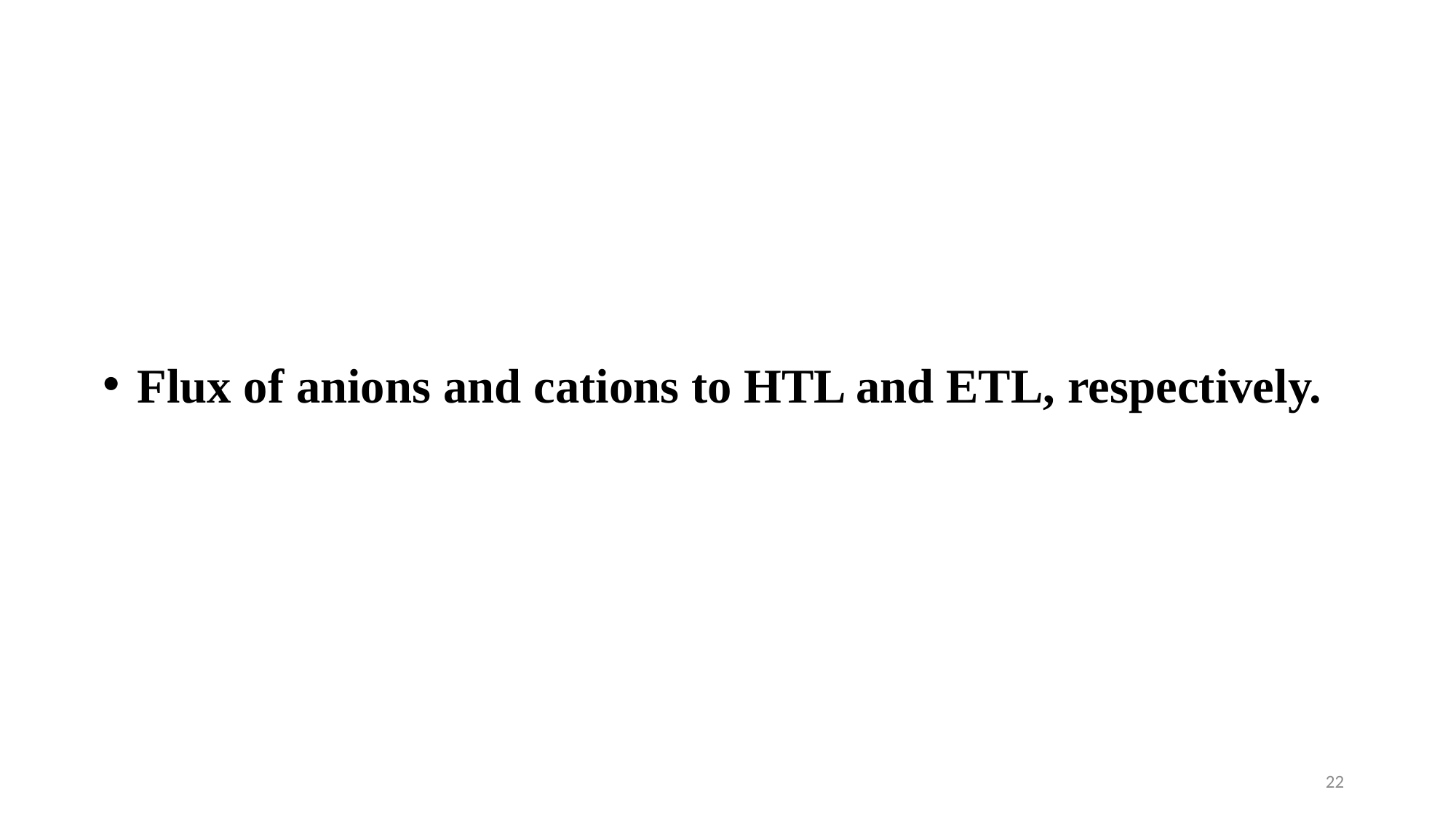

Flux of anions and cations to HTL and ETL, respectively.
22

## Slide 23
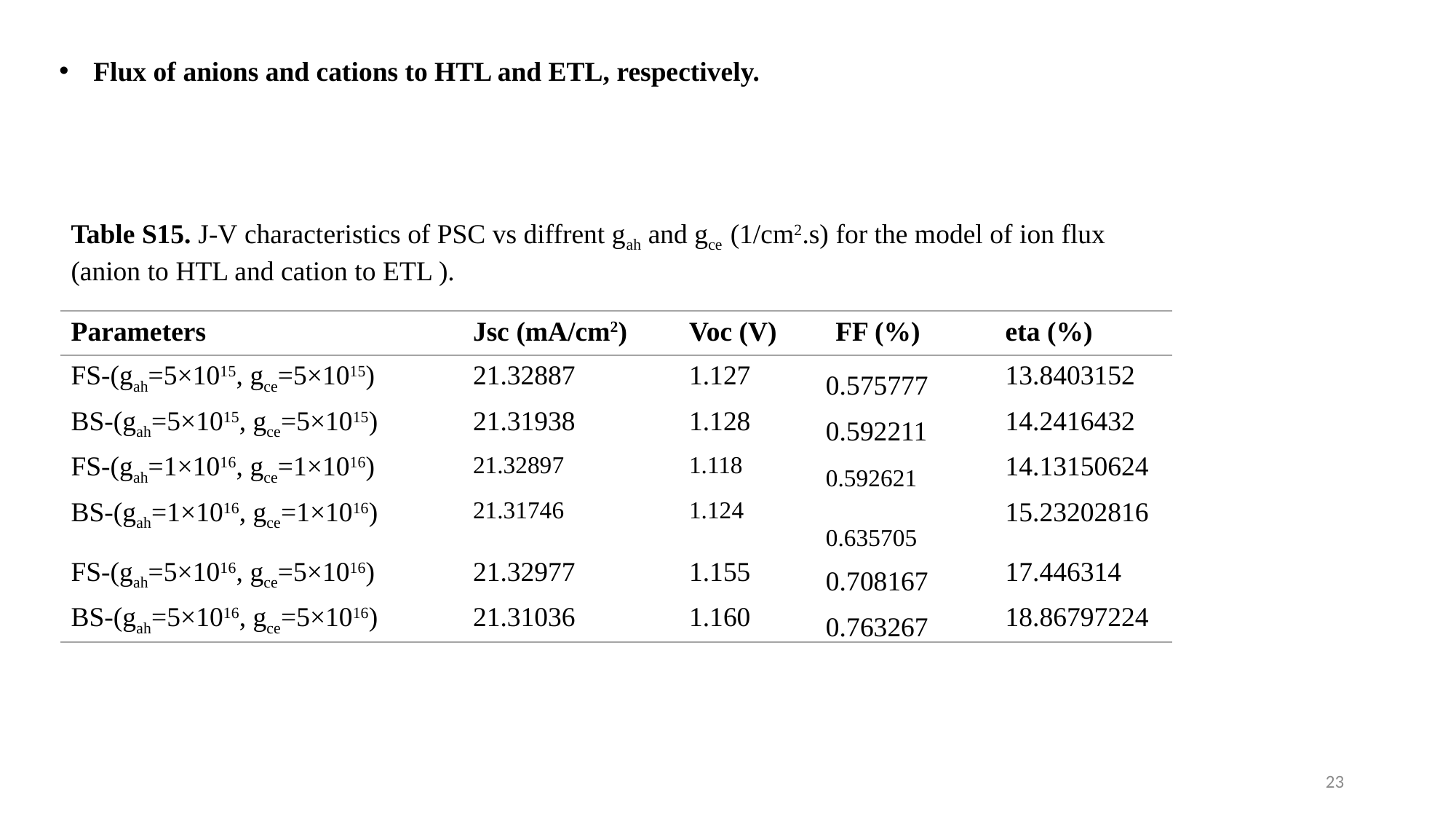

Flux of anions and cations to HTL and ETL, respectively.
Table S15. J-V characteristics of PSC vs diffrent gah and gce (1/cm2.s) for the model of ion flux (anion to HTL and cation to ETL ).
| Parameters | Jsc (mA/cm2) | Voc (V) | FF (%) | eta (%) |
| --- | --- | --- | --- | --- |
| FS-(gah=5×10^15^, gce=5×10^15^) | 21.32887 | 1.127 | 0.575777 | 13.8403152 |
| BS-(gah=5×10^15^, gce=5×10^15^) | 21.31938 | 1.128 | 0.592211 | 14.2416432 |
| FS-(gah=1×10^16^, gce=1×10^16^) | 21.32897 | 1.118 | 0.592621 | 14.13150624 |
| BS-(gah=1×10^16^, gce=1×10^16^) | 21.31746 | 1.124 | 0.635705 | 15.23202816 |
| FS-(gah=5×10^16^, gce=5×10^16^) | 21.32977 | 1.155 | 0.708167 | 17.446314 |
| BS-(gah=5×10^16^, gce=5×10^16^) | 21.31036 | 1.160 | 0.763267 | 18.86797224 |
23

## Slide 24
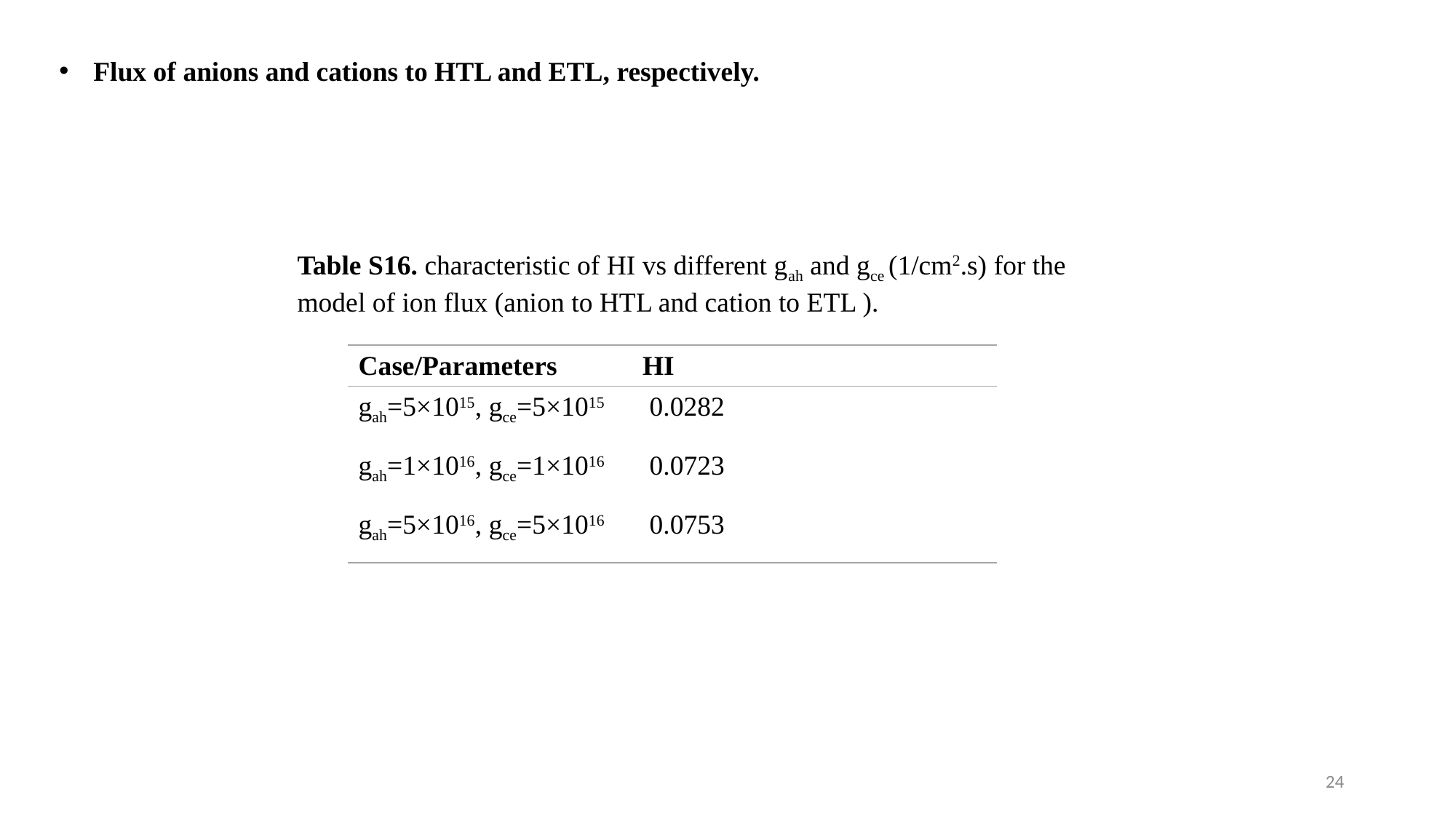

Flux of anions and cations to HTL and ETL, respectively.
Table S16. characteristic of HI vs different gah and gce (1/cm2.s) for the model of ion flux (anion to HTL and cation to ETL ).
| Case/Parameters | HI |
| --- | --- |
| gah=5×10^15^, gce=5×10^15^ | 0.0282 |
| gah=1×10^16^, gce=1×10^16^ | 0.0723 |
| gah=5×10^16^, gce=5×10^16^ | 0.0753 |
24

## Slide 25
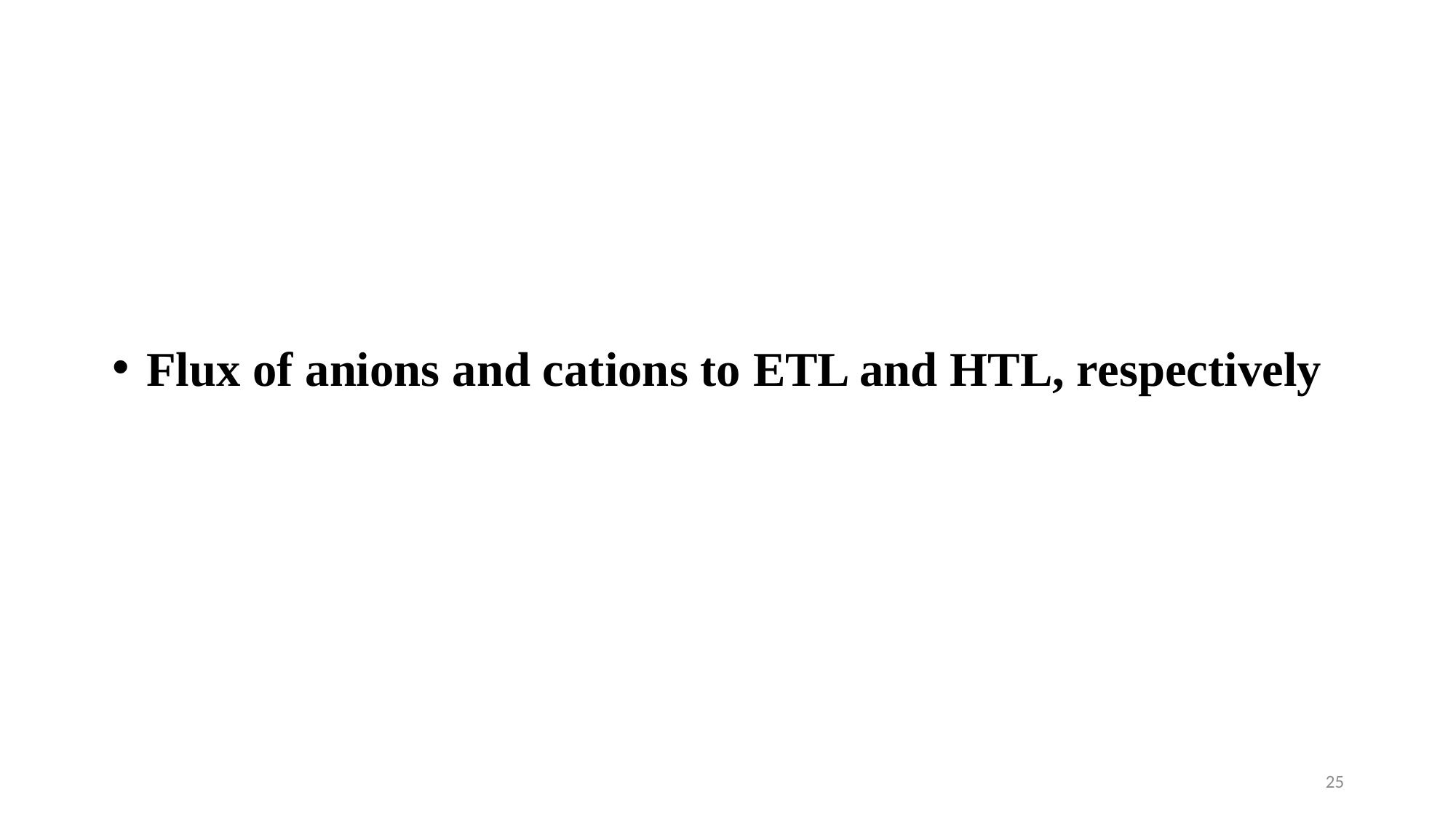

Flux of anions and cations to ETL and HTL, respectively
25

## Slide 26
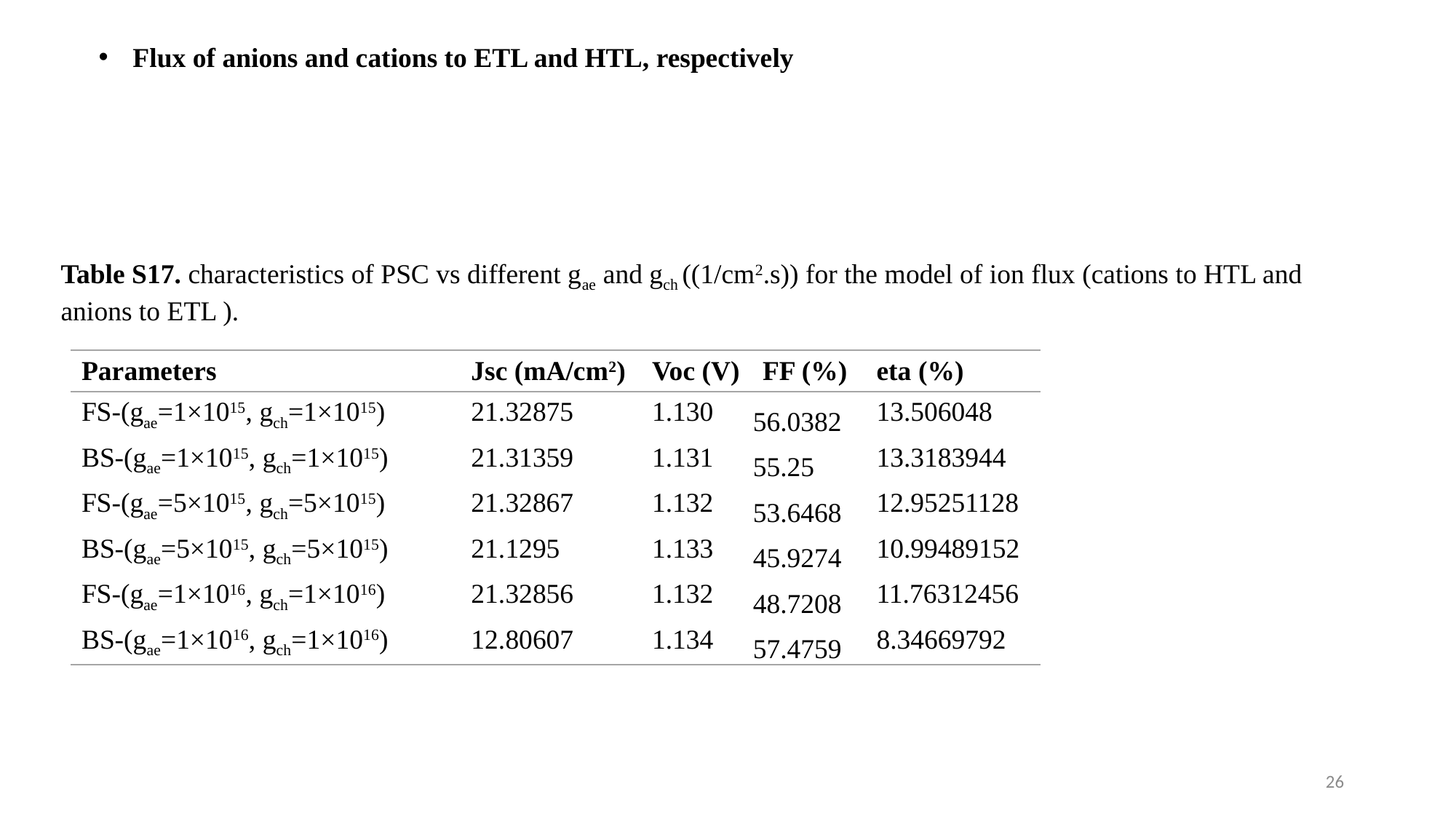

Flux of anions and cations to ETL and HTL, respectively
Table S17. characteristics of PSC vs different gae and gch ((1/cm2.s)) for the model of ion flux (cations to HTL and anions to ETL ).
| Parameters | Jsc (mA/cm2) | Voc (V) | FF (%) | eta (%) |
| --- | --- | --- | --- | --- |
| FS-(gae=1×10^15^, gch=1×10^15^) | 21.32875 | 1.130 | 56.0382 | 13.506048 |
| BS-(gae=1×10^15^, gch=1×10^15^) | 21.31359 | 1.131 | 55.25 | 13.3183944 |
| FS-(gae=5×10^15^, gch=5×10^15^) | 21.32867 | 1.132 | 53.6468 | 12.95251128 |
| BS-(gae=5×10^15^, gch=5×10^15^) | 21.1295 | 1.133 | 45.9274 | 10.99489152 |
| FS-(gae=1×10^16^, gch=1×10^16^) | 21.32856 | 1.132 | 48.7208 | 11.76312456 |
| BS-(gae=1×10^16^, gch=1×10^16^) | 12.80607 | 1.134 | 57.4759 | 8.34669792 |
26

## Slide 27
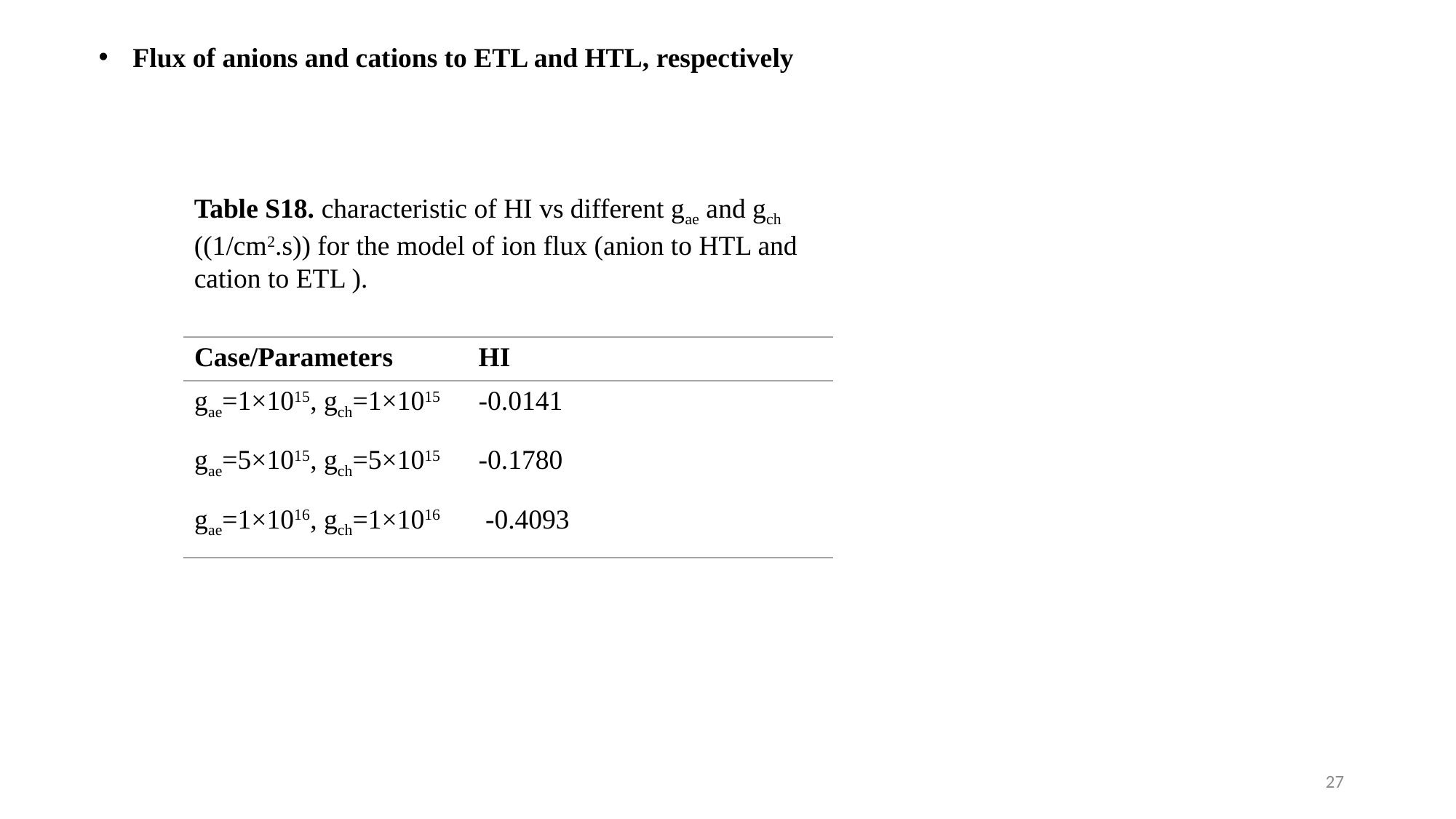

Flux of anions and cations to ETL and HTL, respectively
Table S18. characteristic of HI vs different gae and gch ((1/cm2.s)) for the model of ion flux (anion to HTL and cation to ETL ).
| Case/Parameters | HI |
| --- | --- |
| gae=1×10^15^, gch=1×10^15^ | -0.0141 |
| gae=5×10^15^, gch=5×10^15^ | -0.1780 |
| gae=1×10^16^, gch=1×10^16^ | -0.4093 |
27

## Slide 28
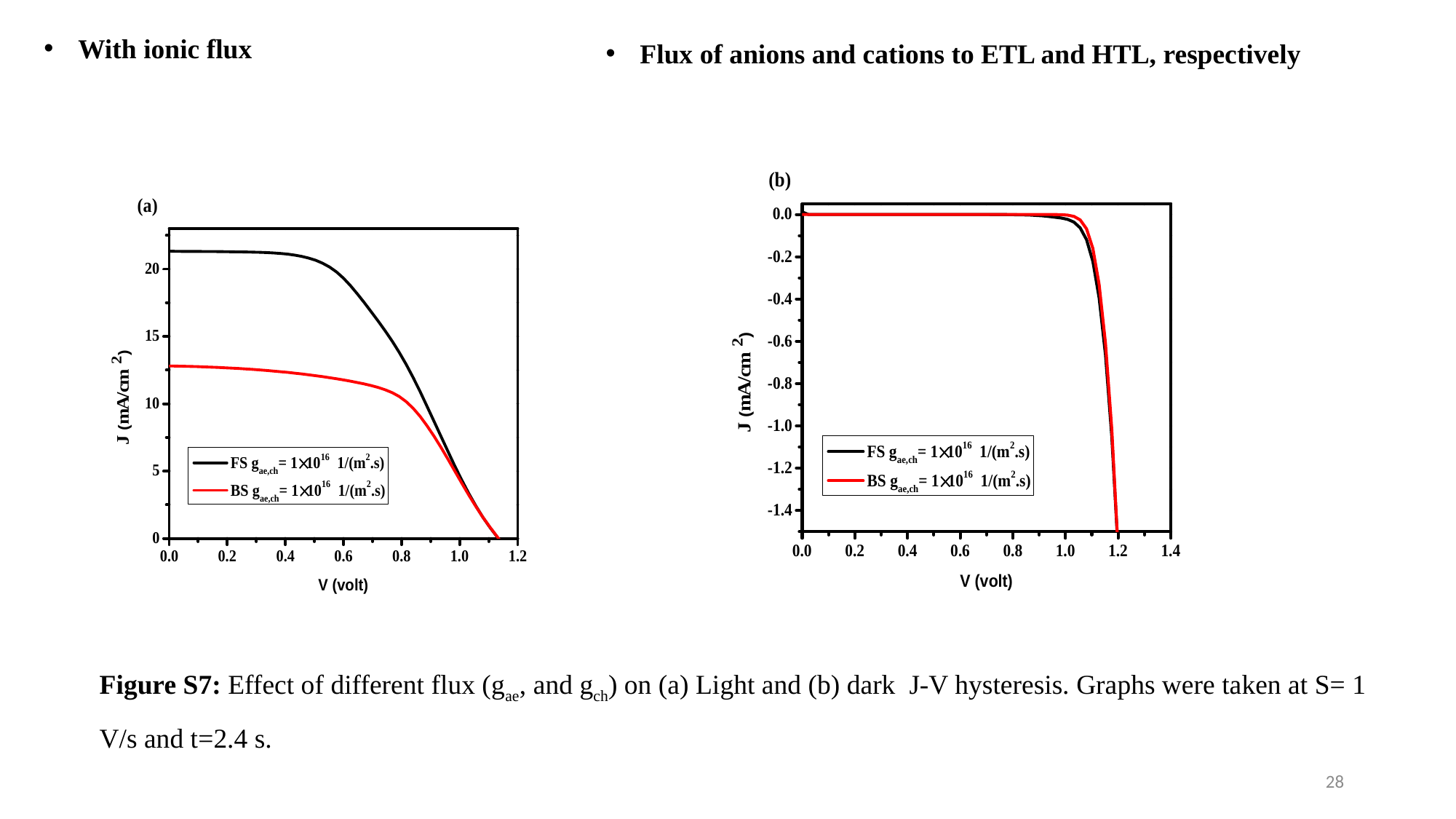

Flux of anions and cations to ETL and HTL, respectively
With ionic flux
Figure S7: Effect of different flux (gae, and gch) on (a) Light and (b) dark J-V hysteresis. Graphs were taken at S= 1 V/s and t=2.4 s.
28

## Slide 29
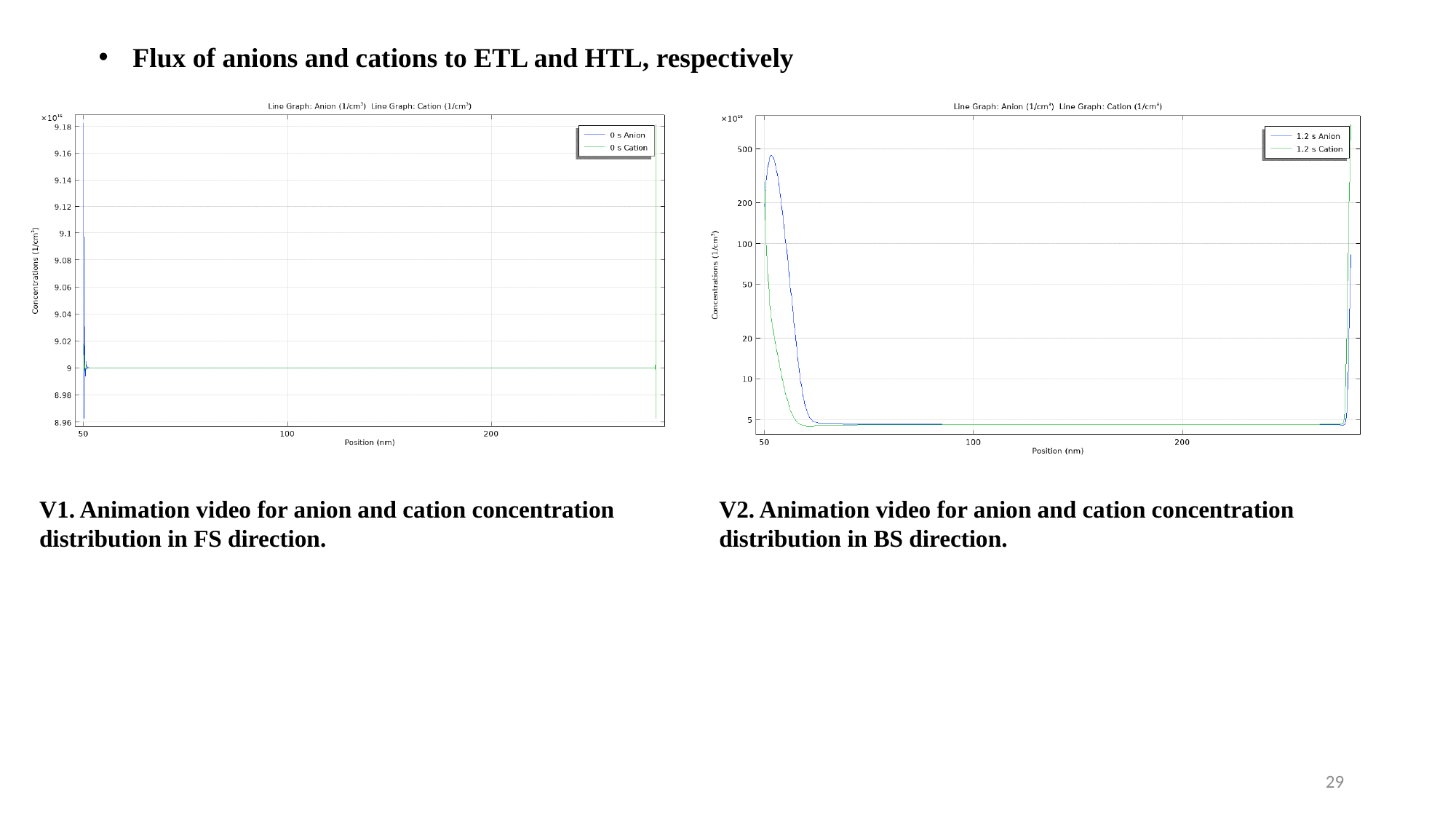

Flux of anions and cations to ETL and HTL, respectively
V2. Animation video for anion and cation concentration distribution in BS direction.
V1. Animation video for anion and cation concentration distribution in FS direction.
29

## Slide 30
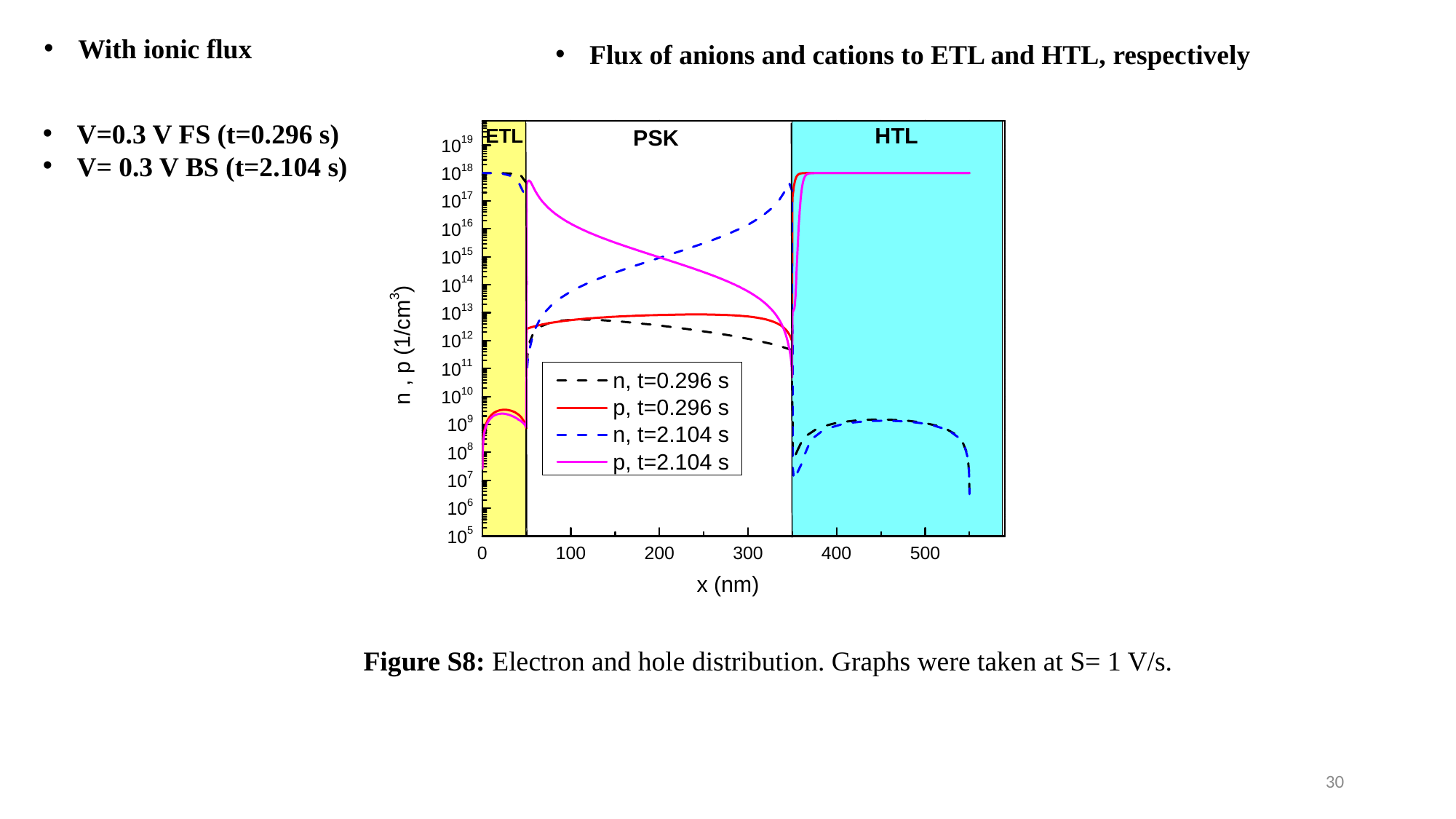

Flux of anions and cations to ETL and HTL, respectively
With ionic flux
V=0.3 V FS (t=0.296 s)
V= 0.3 V BS (t=2.104 s)
Figure S8: Electron and hole distribution. Graphs were taken at S= 1 V/s.
30

## Slide 31
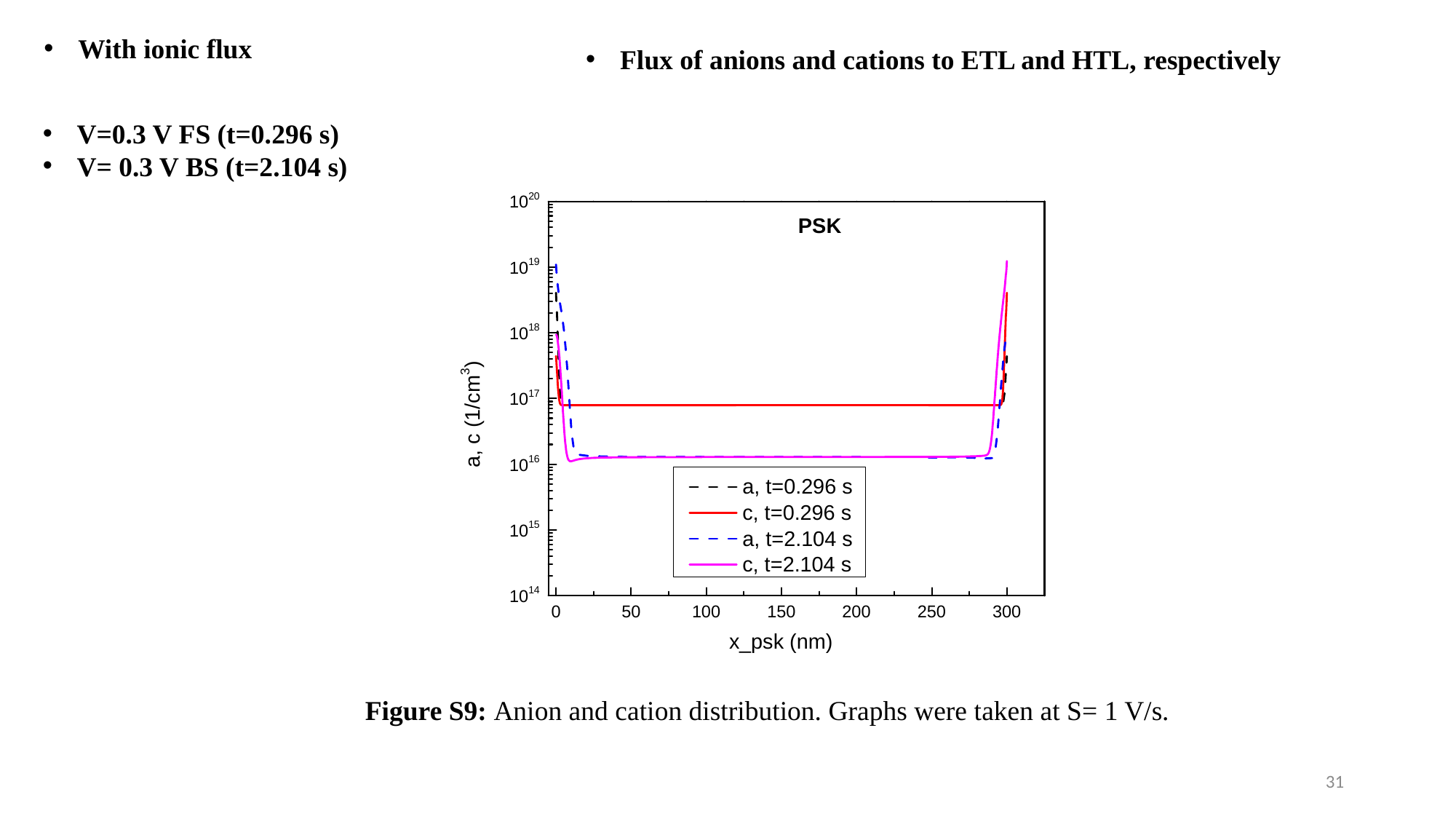

Flux of anions and cations to ETL and HTL, respectively
With ionic flux
V=0.3 V FS (t=0.296 s)
V= 0.3 V BS (t=2.104 s)
Figure S9: Anion and cation distribution. Graphs were taken at S= 1 V/s.
31

## Slide 32
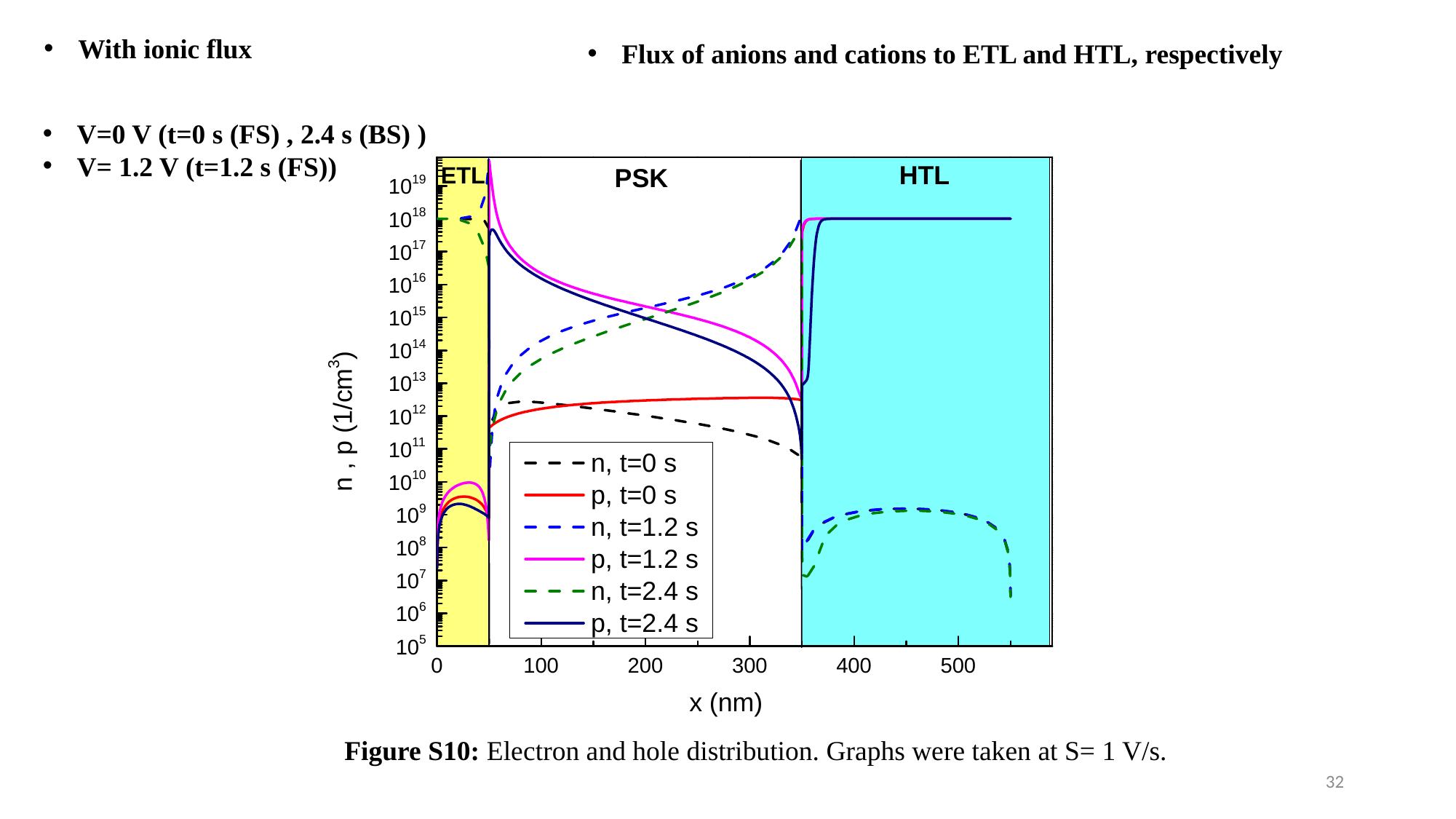

Flux of anions and cations to ETL and HTL, respectively
With ionic flux
V=0 V (t=0 s (FS) , 2.4 s (BS) )
V= 1.2 V (t=1.2 s (FS))
Figure S10: Electron and hole distribution. Graphs were taken at S= 1 V/s.
32

## Slide 33
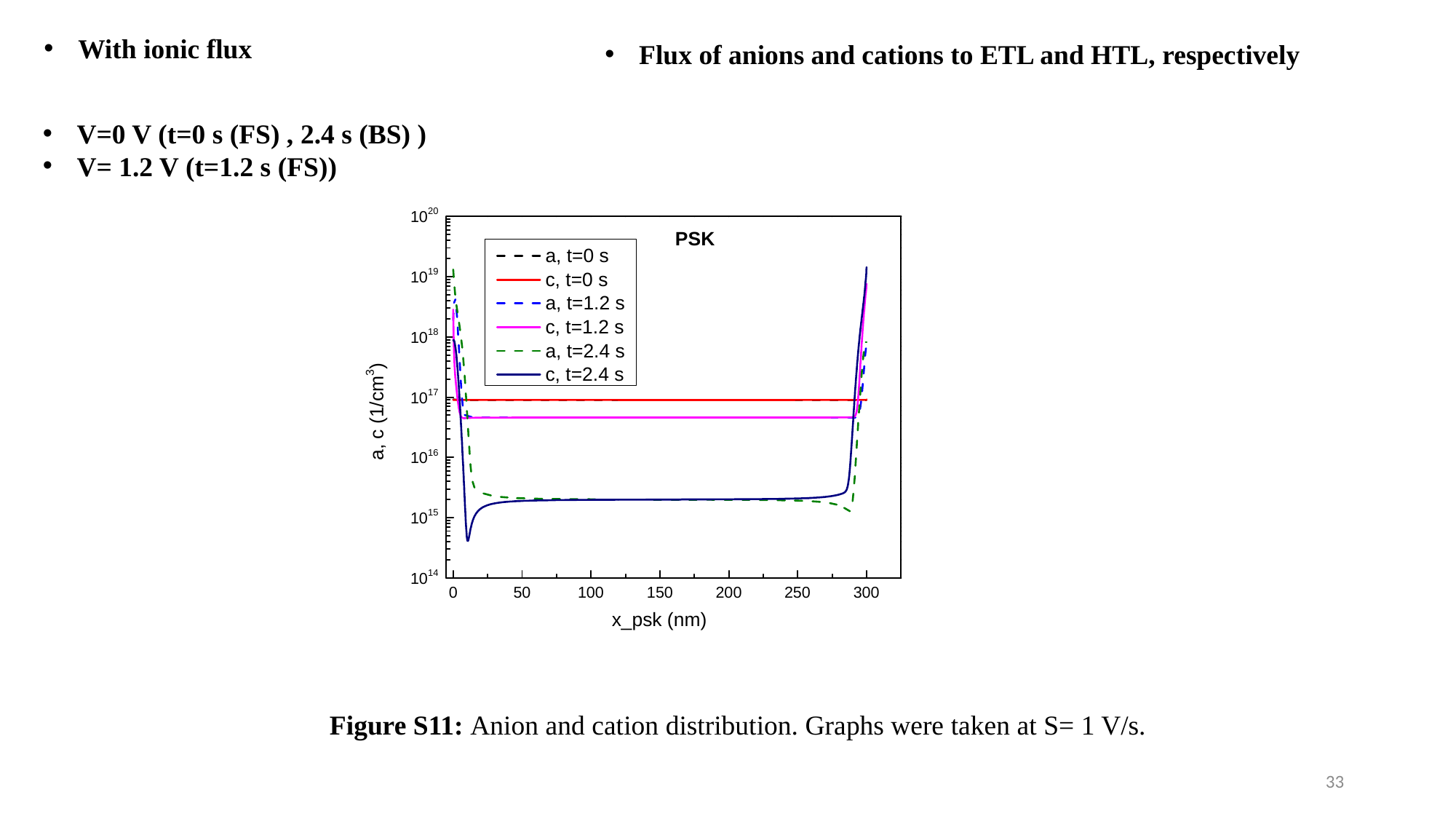

Flux of anions and cations to ETL and HTL, respectively
With ionic flux
V=0 V (t=0 s (FS) , 2.4 s (BS) )
V= 1.2 V (t=1.2 s (FS))
Figure S11: Anion and cation distribution. Graphs were taken at S= 1 V/s.
33

## Slide 34
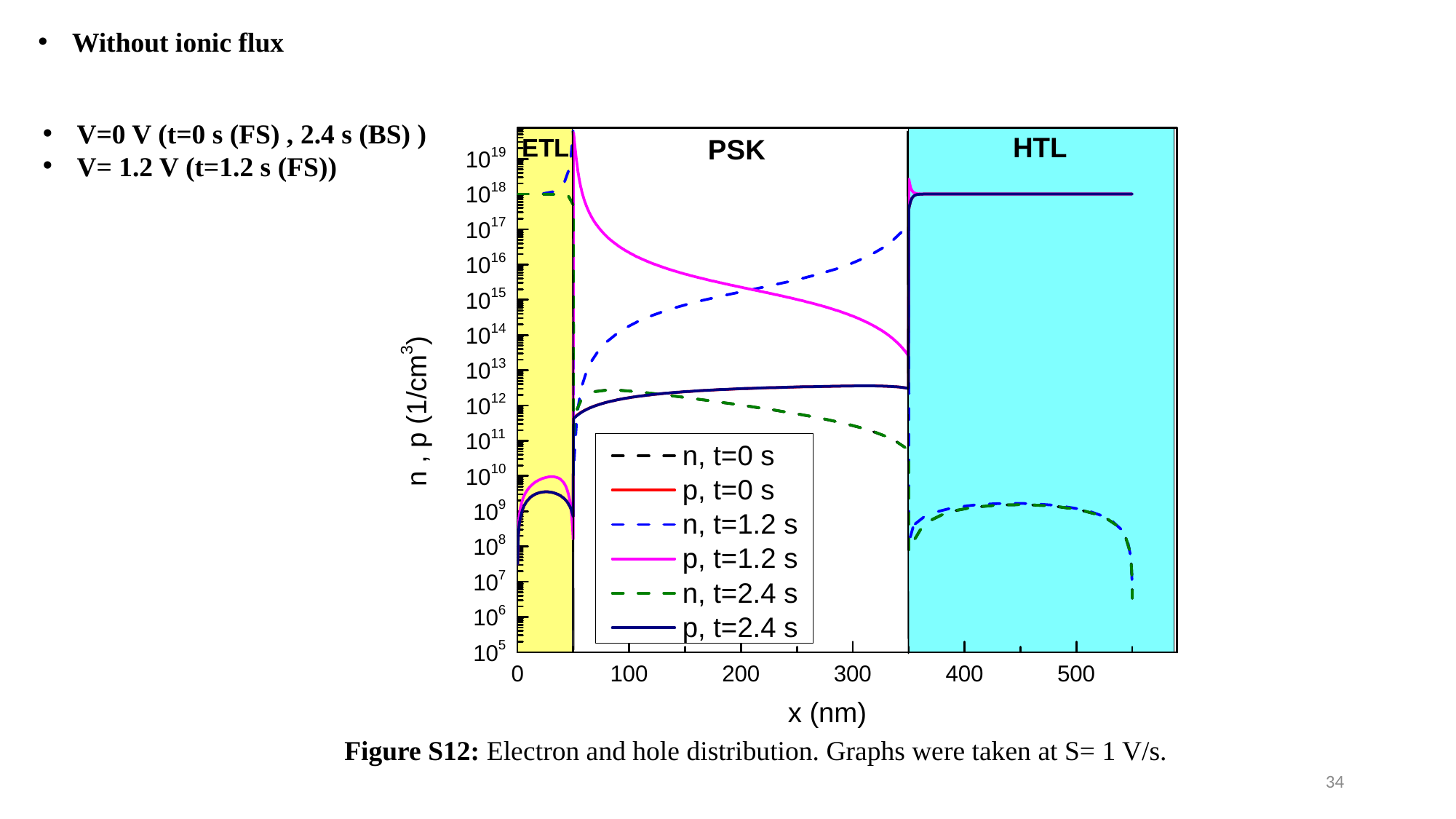

Without ionic flux
V=0 V (t=0 s (FS) , 2.4 s (BS) )
V= 1.2 V (t=1.2 s (FS))
Figure S12: Electron and hole distribution. Graphs were taken at S= 1 V/s.
34

## Slide 35
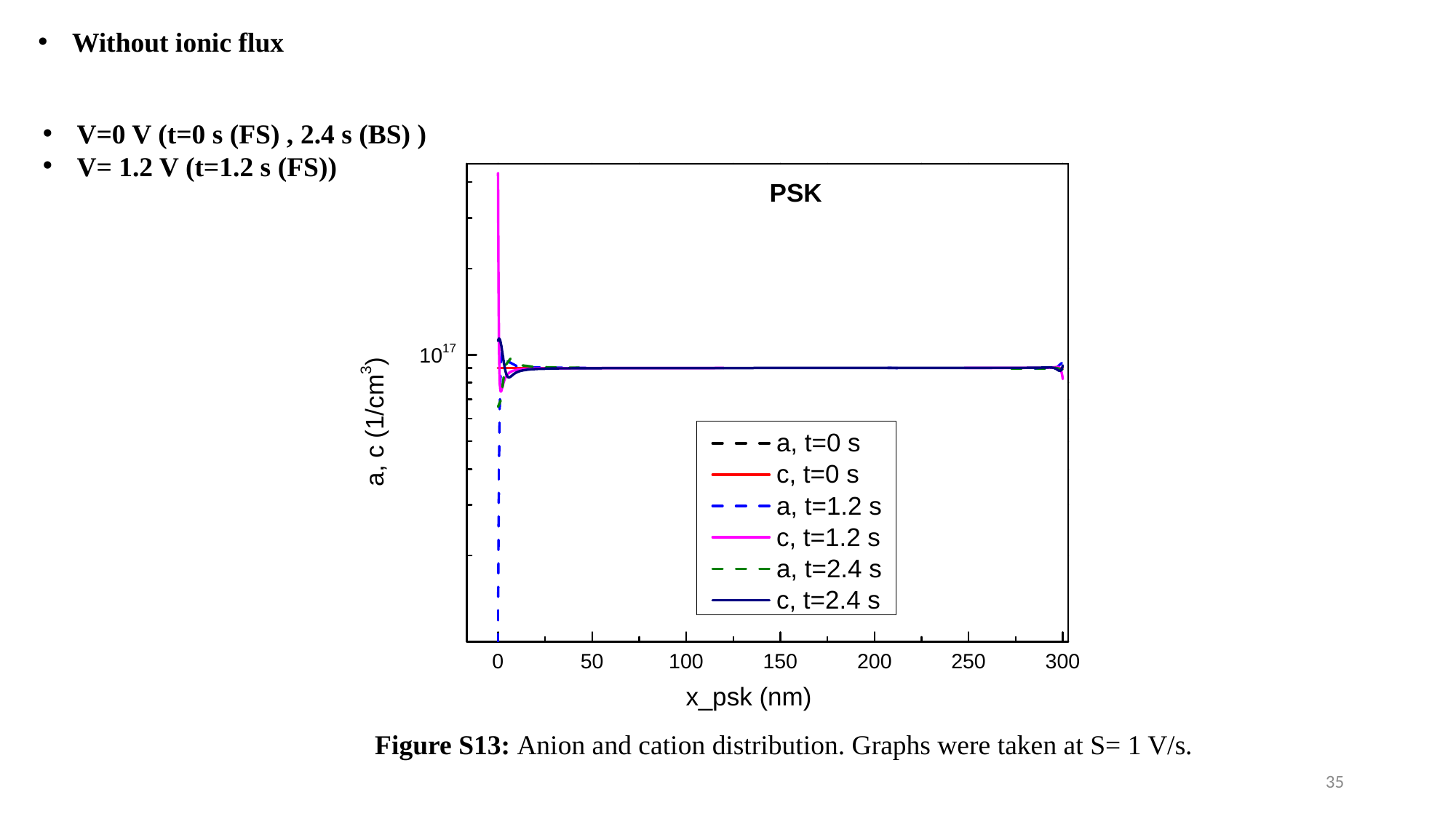

Without ionic flux
V=0 V (t=0 s (FS) , 2.4 s (BS) )
V= 1.2 V (t=1.2 s (FS))
Figure S13: Anion and cation distribution. Graphs were taken at S= 1 V/s.
35
