# Supplementary figures and images for: Comprehensive study of anomalous hysteresis behavior in perovskite-based solar cells
Source: Sci Rep. 2022 Sep 1;12:14916. doi: 10.1038/s41598-022-19194-5 (PMC9436975; doi:10.1038/s41598-022-19194-5)

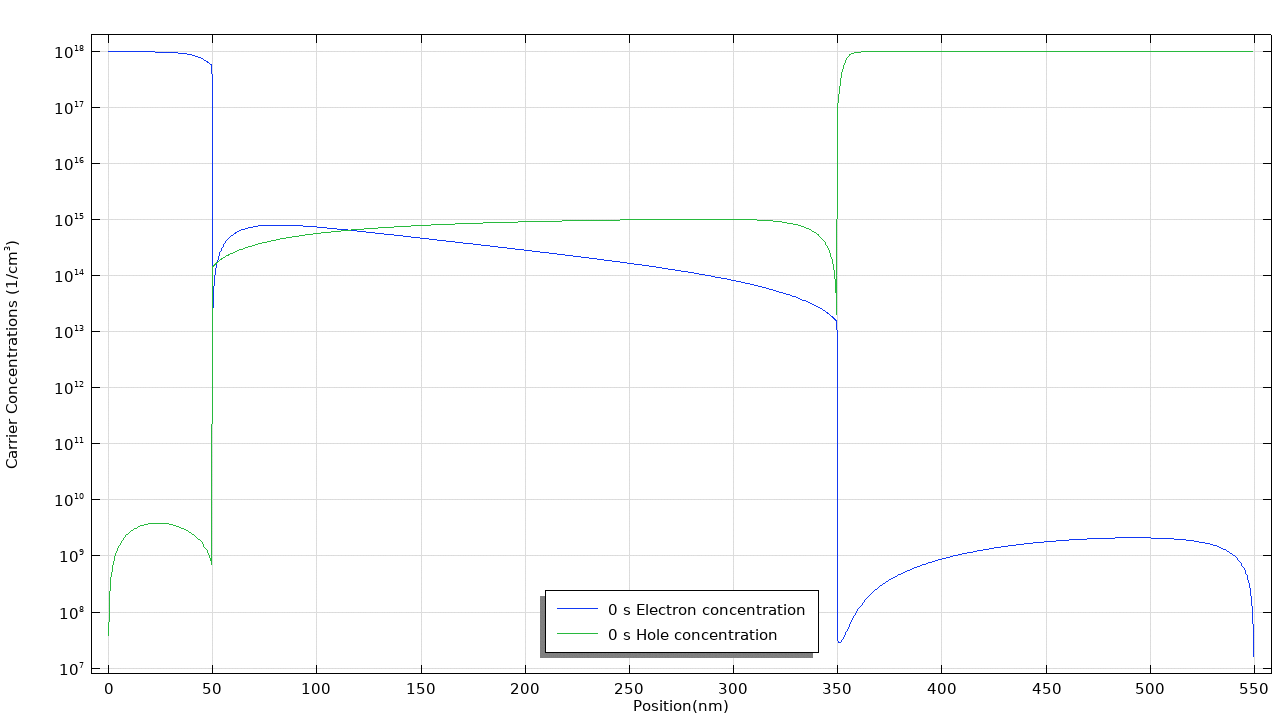

Supplement: Supplementary file 2 — Supplementary Information 2. [file 41598_2022_19194_MOESM2_ESM.gif]

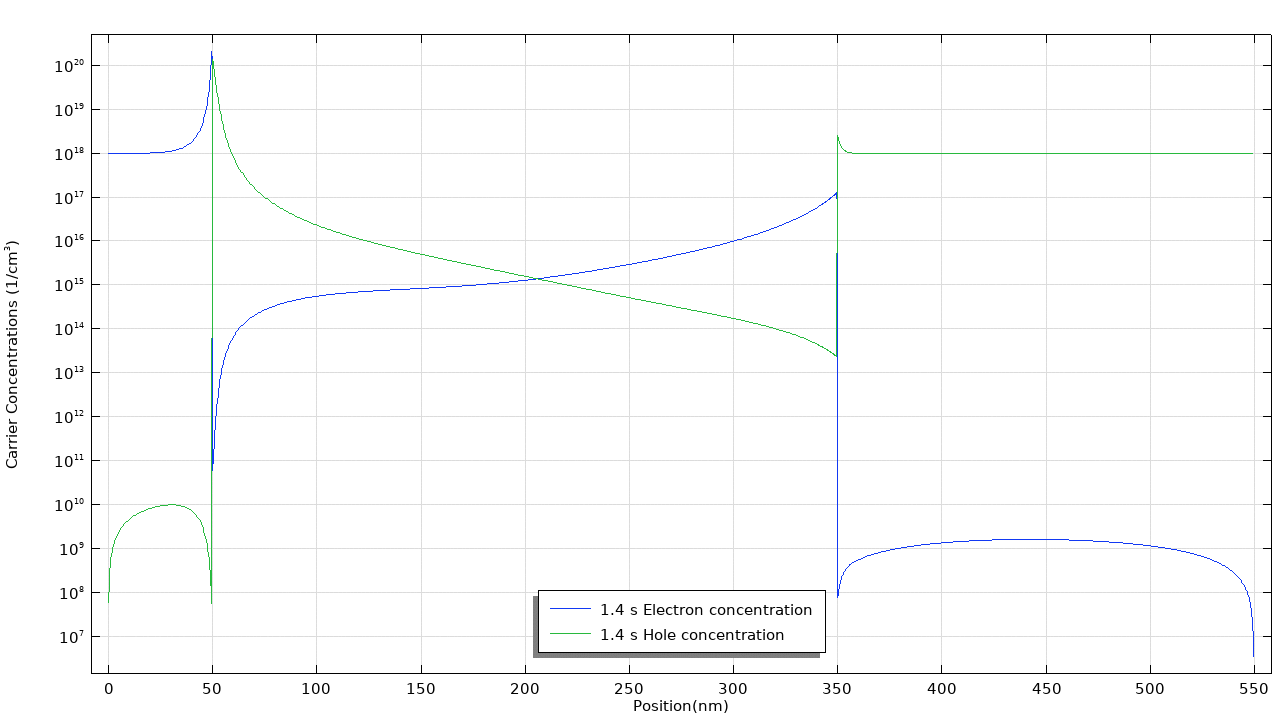

Supplement: Supplementary file 3 — Supplementary Information 3. [file 41598_2022_19194_MOESM3_ESM.gif]
